# Antitumor activity of the aurora a selective kinase inhibitor, alisertib, against preclinical models of colorectal cancer

**DOI:** 10.18632/oncotarget.10366

**Published:** 2016-07-01

**Authors:** Todd M. Pitts, Erica L. Bradshaw, Stacey M. Bagby, Stephanie L. Hyatt, Heather M. Selby, Anna Spreafico, John J. Tentler, Kelly McPhillips, Peter J. Klauck, Anna Capasso, Jennifer R. Diamond, S. Lindsey Davis, Aik Choon Tan, John J. Arcaroli, Alicia Purkey, Wells A. Messersmith, Jeffery A. Ecsedy, S. Gail Eckhardt

**Affiliations:** ^1^ Division of Medical Oncology, School of Medicine, University of Colorado, Anschutz Medical Campus, Aurora, CO, USA; ^2^ Department of Pharmaceutical Sciences, Skaggs School of Pharmacy and Pharmaceutical Sciences, University of Colorado, Anschutz Medical Campus, Aurora, CO, USA; ^3^ University of Colorado Cancer Center, University of Colorado Anschutz Medical Campus, Aurora, CO, USA; ^4^ Millennium Pharmaceuticals, Inc., a wholly owned subsidiary of Takeda Pharmaceutical Company Limited, Cambridge, MA, USA; ^5^ Takeda California, San Diego, CA, USA

**Keywords:** colorectal cancer, aurora kinase a

## Abstract

**Background:**

The Aurora kinases are a family of serine/threonine kinases comprised of Aurora A, B, and C which execute critical steps in mitotic and meiotic progression. Alisertib (MLN8237) is an investigational Aurora A selective inhibitor that has demonstrated activity against a wide variety of tumor types *in vitro* and *in vivo*, including CRC.

**Results:**

CRC cell lines demonstrated varying sensitivity to alisertib with IC_50_ values ranging from 0.06 to > 5 umol/L. Following exposure to alisertib we observed a decrease in pAurora A, B and C in four CRC cell lines. We also observed an increase in p53 and p21 in a sensitive p53 wildtype cell line in contrast to the p53 mutant cell line or the resistant cell lines. The addition of alisertib to standard CRC treatments demonstrated improvement over single agent arms; however, the benefit was largely less than additive, but not antagonistic.

**Methods:**

Forty-seven CRC cell lines were exposed to alisertib and IC_50_s were calculated. Twenty-one PDX models were treated with alisertib and the Tumor Growth Inhibition Index was assessed. Additionally, 5 KRAS wildtype and mutant PDX models were treated with alisertib as single agent or in combination with cetuximab or irinotecan, respectively.

**Conclusion:**

Alisertib demonstrated anti-proliferative effects against CRC cell lines and PDX models. Our data suggest that the addition of alisertib to standard therapies in colorectal cancer if pursued clinically, will require further investigation of patient selection strategies and these combinations may facilitate future clinical studies.

## INTRODUCTION

Classical drug development in oncology has often focused on targeting the DNA replication machinery in cancer cells. Of late, the Aurora kinase family has been attracting interest as its members carry out essential roles in centrosome maturation and chromosome segregation [1]. Aurora kinases are a family of serine-threonine protein kinases that are involved in spindle pole organization and mitotic progression [2]. The three known Aurora kinases in humans (A, B, C) are distinguished by their specific functions during mitosis [3]. Aurora kinase A is upregulated in the G2 phase of the cell cycle and localizes to the centrosomes during interphase and to both spindle poles and spindle microtubules during early mitosis. Aurora kinase B is expressed in proliferating cells during G2 and mitosis and is essential for chromosome condensation, biorientation and cytokinesis. Lastly, Aurora kinase C has been identified as a chromosomal passenger protein and resembles Aurora B in location and function [4, 5].

Many human malignancies, including those of the breast, colon, pancreas, and prostate have aberrant expression of Aurora kinase A. Overexpression of Aurora A is associated with with a poor prognosis and may be associated with resistance to gefitinib, taxol, and cisplatin in various cancers [6–8]. Aurora kinase A has been shown to directly phosphorylate p53 at Ser315 and inactivate it by enhancing its proteolytic degradation to cause a phenotype similar to loss of function of p53. This interaction is thought to also cause the aneuploidy observed with Aurora kinase A overexpression [9–12]. Aurora kinase B has also been implicated in carcinogenesis. Cells that overexpress Aurora kinase B demonstrate elevated levels of phosphorylated histone H3 and defects in cytokinesis. In some colorectal cancer cell lines, increased levels of phosphorylated histone H3 correlate with overexpression of Aurora kinase B [13].

Alisertib (MLN8237) is a selective Aurora kinase A inhibitor that is currently undergoing Phase I-III clinical development (www.clinicaltrials.gov). Alisertib demonstrates a 300-fold higher selectivity to Aurora kinase A over B in *in vitro* assays and 200-fold higher selectivity in the HCT116 colorectal cancer cell line [14]. Preclinically, alisertib has demonstrated efficacy in numerous tumor types. In multiple myeloma cells treated with alisertib, mitotic spindle abnormalities and mitotic accumulation was observed, this led to inhibition of proliferation, induction of apoptosis and senescence. This treatment also resulted in upregulation of p53, p21 and p27. Furthermore, in triple negative breast cancer alisertib demonstrated antiproliferative effects regardless of subtype, however there was a trend whereby increased p53 mRNA expression associated with increased sensitivity to alisertib [15]. In addition, in combination with dexamethasone, doxorubicin, or bortezomib, alisertib induced synergistic./additive activity *in vitro* [16]. Against esophageal cancer models, alisertib demonstrated antitumor activity as a single agent and in combination with cisplatin *in vitro* and *in vivo* [17]. Likewise against bladder cancer cells, alisertib induced cell-cycle arrest, aneuploidy, and mitotic spindle failure as well as synergistic anti-tumor effects with gemcitabine or paclitaxel [18]. Clinically, alisertib has demonstrated early evidence of efficacy in multiple tumor types. In a Phase I clinical trial in advanced solid tumors, 37% of patients achieved stable disease with a median duration of 7.3 months. The stable disease was durable for more than 6 months in 6 patients and for more than 1 year in 4 patients diagnosed with colorectal cancer (CRC), chondrosarcoma, leiomyosarcoma, and liposarcoma [19]. More recently, a Phase II clinical trial in patients with breast, small-cell lung, non-small-cell lung, head and neck, and gastro-esophageal cancers alisertib demonstrated varying benefit as a single agent. In breast cancer patients, an 18% objective response was observed with hormone receptor-positive and HER-2 positive subgroups demonstrating a better response. The small-cell lung cancer cohort had an objective response of 21%, however the non-small-cell cohort only a 4% objective response was observed. In the head and neck, and gastro-esophageal cohorts a 9% objective response was observed [20].

Based on the novel mechanism of action and its preclinical and early clinical activity, we sought to assess the antitumor activity of alisertib against colorectal cancer models *in vitro* and in patient derived xenograft models (PDX), as a single agent and in combination with standard therapies.

## RESULTS

### Anti-proliferative effects of alisertib against colorectal cancer cell lines

**Figure 1:**
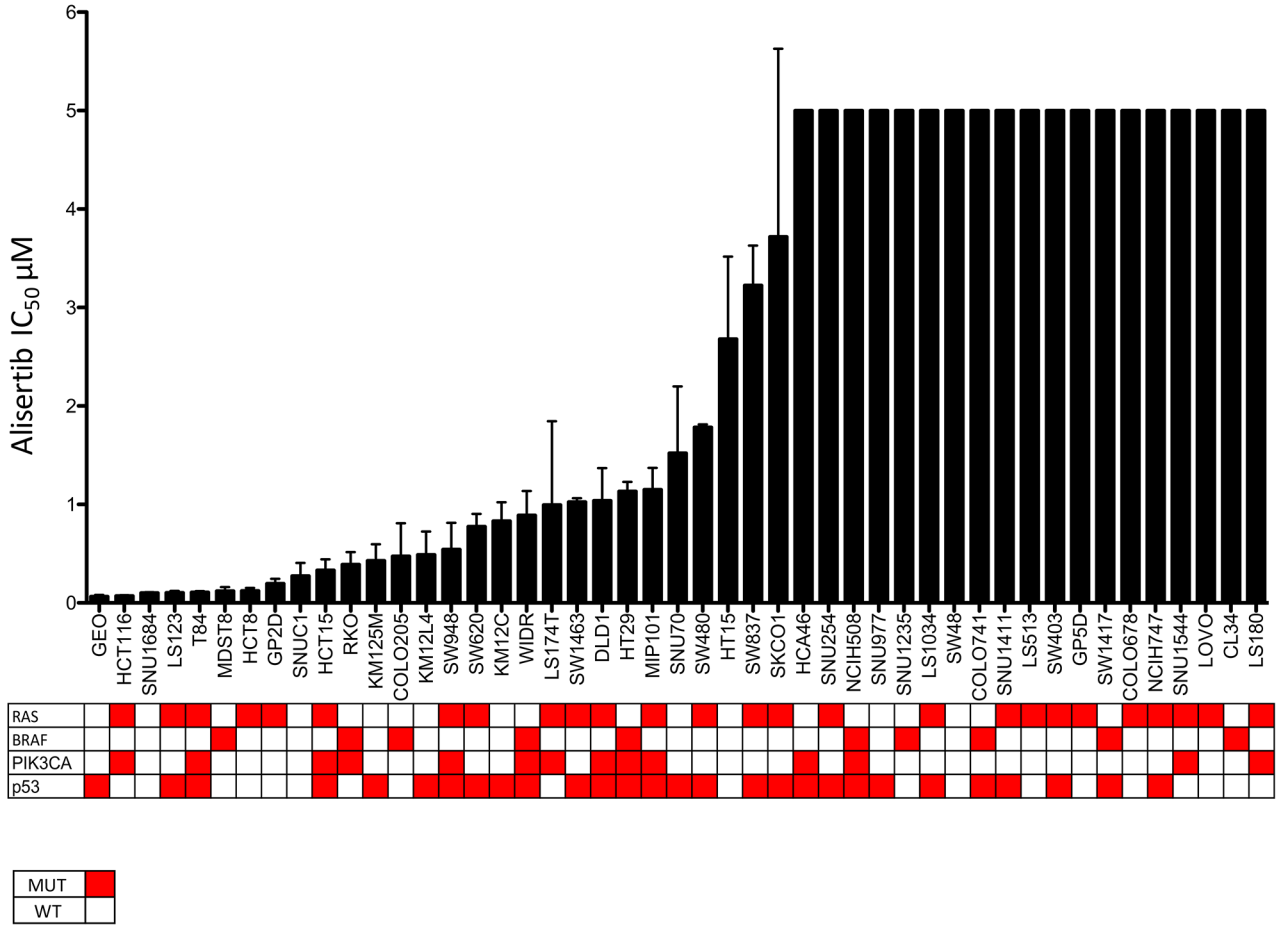
CRC cell lines exposed to alisertiv to establish their IC_50s_ Cell lines were treated with increasing concentraitons of alisertib and IC_50_ values were calculated. There was a broad range of sensitivity to the agent.

We initially sought to determine the relative sensitivity of our CRC panel to alisertib using the CyQuant proliferation assay. As depicted in Figure 1 there was a wide range of IC_50_s among the CRC cell lines that did not correlate with mutational status of KRAS, BRAF, PIK3CA, or p53. However, Gene Set Enrichment Analysis (GSEA) did show that the cell cycle pathway was upregulated in the more sensitive cell lines, when compared to resistant (data not shown). Core genes in this pathway include CDK1, CDK4, CDK6, CyclinD1, and PLK1. Two sensitive (HCT116, LS123) and two resistant (GP5D, COLO678) CRC cell lines, were chosen for further *in vitro* analysis.

### Assessment of alisertib targets and downstream effectors by immunoblotting in CRC cell lines

**Figure 2:**
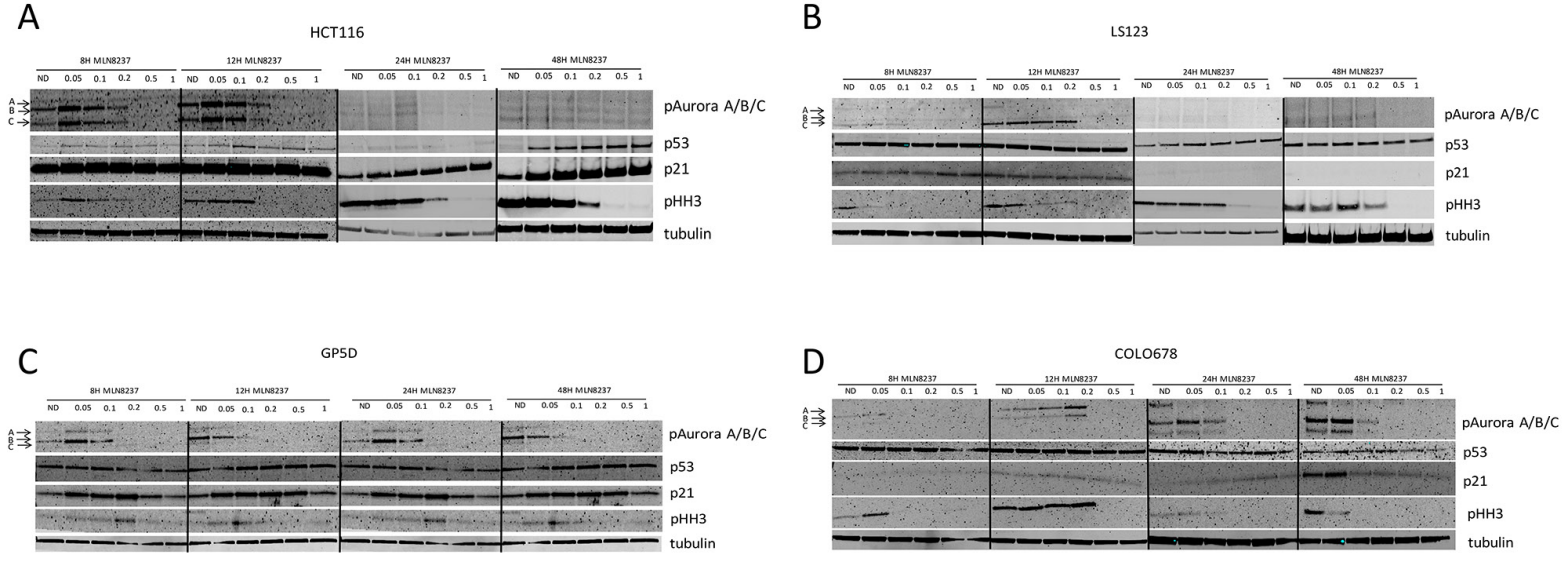
Effect of alisertib on downstream effectors **(A** and **B).** Two sensitive and **(C** and **D).** two resistant CRC cell lines were exposed to alisertib for 8, 12, 24 and 48 hours. Western blot analysis of proteins following alisertib treatment. In general, regardless of sensitivity to alisertib a decrease of Aurora kinases were observed.

To determine the effects of alisertib on CRC cell lines, immunoblotting was performed for selected proteins at 8, 12, 24 and 48 hours. As depicted in Figure 2, we observed a decrease of phosphorylated A, B, and C regardless of sensitivity. However, in the p53 mutant CRC cell line, LS123, it appears that higher doses of alisertib are needed to see target effects. Previous reports have indicated that Aurora A can functionally interact and inactivate p53 to modulate its expression levels [10]. Given this we sought to assess the effects of alisertib on p53 levels following drug exposure. In the p53 wild type cell line, HCT116, p53 expression was negligibly increased at 24 hours while clearly induced at 8, 12, and 48 hours and associated with p21 induction at the later time points, an indication of cell cycle arrest. By contrast, in the p53 mutant (R175H) CRC cell line, LS123, a minor increase in p53 was only observed at 24 hours and there was no associated p21 induction. Conversely, no substantial effect on p53 was observed in the two resistant CRC cell lines. Since both Aurora A and B impact the phosphorylation status of histone H3 we evaluated the effects of alisertib on the selected CRC cell lines. In three out of the four cell lines tested (HCT116, LS123, and COLO678), a dramatic decrease in phosphorylated histone H3 was detected, indicating inhibition of Aurora B and less Aurora A selectivity by alisertib. This decrease appears to be cell line and time dependent.

### Cell cycle effects of alisertib in CRC cell lines

**Figure 3:**
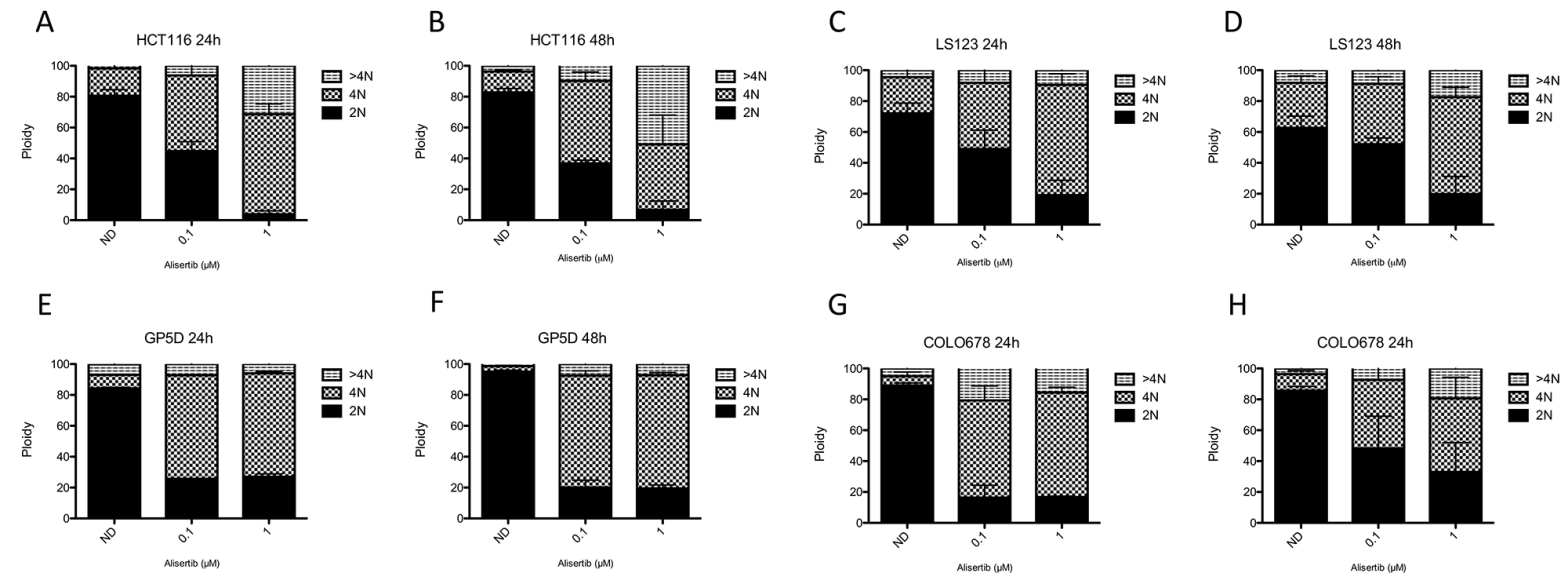
Cell cycle and ploidy analysis **(A-D).** Two sensitive and **(E-H).** two resistant CRC cell lines were treated for 24 and 48 hours to alisertib, stained with propidium iodide and flow analysis was performed. All four cell lines demonstrated a G2/M arrest following treatment with alisertib at 24 and 48 hours.

Flow cytometry was performed to assess the effects of alisertib on cell cycle dynamics/ploidy. CRC cell lines were treated with alisertib (0.1 μmol/L and 1 μmol/L) for 24 and 48 hours. As depicted in Figure 3 (A, C, E, G) following 24 hours of treatment we observed an increase of tetraploid cells, indicating an arrest in the G2/M phase in all four CRC cell lines. Following 24 hours of treatment, the proportion of tetraploid (G2/M arrested) cells increased from 19% with no treatment to 70% with 1 μmol/L of alisertib in the HCT116, 28% to 65% in the LS123, 27% to 67% in the GP5D, and 17% to 67% in the COLO678. This is indicative of the effects alisertib has on both Aurora A and Aurora B at this concentration. Alisertib treatment induced aneuploidy in three of the four CRC cell lines (HCT116, LS123, and COLO678), but the greatest induction was noted in the HCT116 cell line. A similar effect was observed following 48 hours (Figure 3B, 3D, 3F, and 3H) of exposure, however in the HCT116 CRC cell line, a higher percentage of cells exhibit >4N as compared to 24 hour treatment. This was not observed in the LS123, GP5D, and COLO678 cell lines, where there was no significant difference between the 24 and 48 hour exposures.

### Effects of alisertib on apoptosis in CRC cell lines

**Figure 4:**
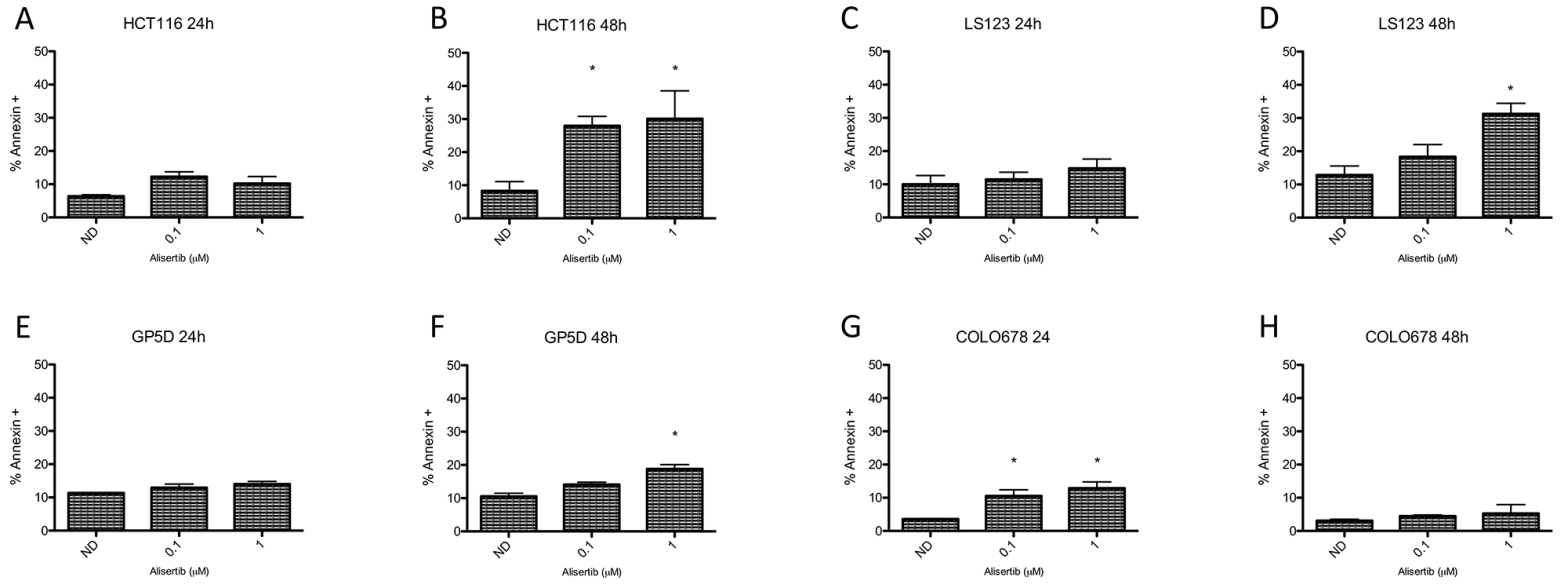
Assessment of apoptosis by annexin V staining **(A-D).** Two sensitive and **(E-H).** two resistant CRC cell lines were treated for 24 and 48 hours, statined with annexin V and propidium iodide and flow analysis was performed. The two sensitive cell lines (HCT116 and LS123) demonstrated a greater increase of apoptosis compared to the resistant lines (GP5D and COLO678).

To determine the effects of alisertib on induction of apoptosis, HCT116, LS123, GP5D, and COLO678 were exposed to the compound for 24 and 48 hours and cells were stained for Annexin V and propidium iodide, followed by flow cytometry analysis. As depicted in Figure 4, there was little induction of apoptosis at 24 hours (Figure 4A, 4C, 4E, and 4G), whereas at 48 hours (Figure 4B, 4D, 4F, and 4G) there was a statistically significant increase in apoptosis in HCT116 cells at 0.1 and 1 μmol/L (p<0.05) and in the LS123 and GP5D cells at 1 μmol/L (p<0.05).

### Efficacy of alisertib in patient derived xenograft models

**Figure 5:**
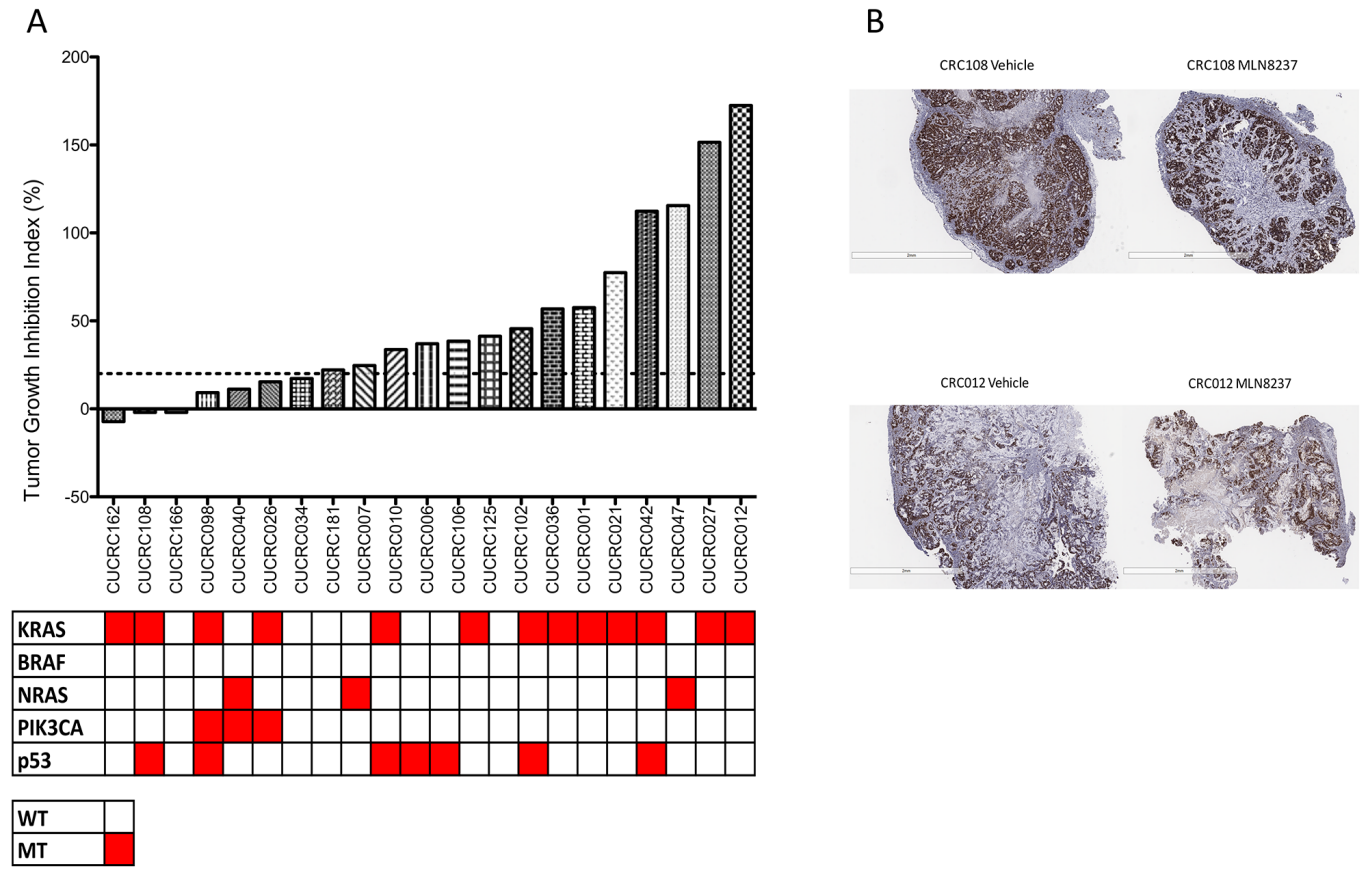
Tumor growth inhibition index (TGII) of all PDX models: TGII = treated over control, thus lower numbers indicate greater tumor reduction **A.** Seven explants were found to be sensitive to alisertib (TGII<20%). **B.** Representitive depiction of Ki67 staining in one responsive and one non-responsive PDX.

To assess the efficacy of alisertib in mouse models of colorectal cancer, twenty-one CRC PDX models were treated with 30 mg/kg alisertib for at least 28 days and tumor growth inhibition index was calculated. As depicted in Figure 5A, there was a wide range of responses to alisertib treatment. Three models, CUCRC162, CUCRC108, and CUCRC166 exhibited minor regression (TGII<0). Four additional models, CUCRC098, CUCRC040, CUCRC026, and CUCRC034 demonstrated a TGII of less than 20% TGII which was defined as a responder to alisertib. In all, seven out of the twenty-one models (33.3%) were deemed responsive to single agent alisertib. This is correlated by a decrease in Ki67 staining and an increase in necrosis in a responsive tumor compared to a non-responsive tumor (Figure 5B). There was no correlation between response to alisertib and mutational status of KRAS, BRAF, NRAS, PIK3CA, and p53.

### Alisertib in combination with standard of care in PDX models

**Figure 6:**
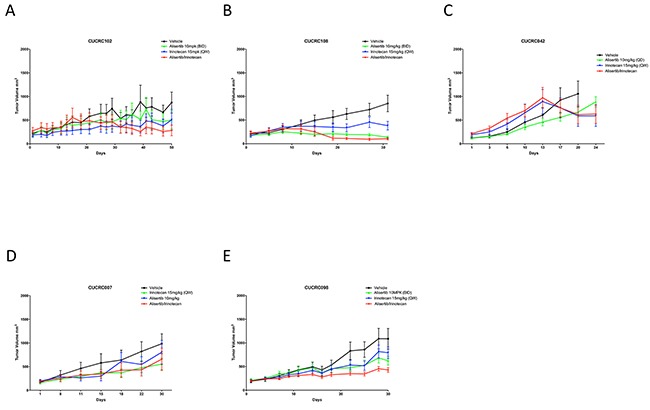
Growth curves of KRAS mutant PDX models treated with alisertib, irinotecan, or the combinaiton: Five CRC PDX models were treated with vehicle, alisertib (10 mg/kg, QD) alone, irinotecan (15 mg/kg, QW) alone, or the combination for at least 20 days

**Figure 7:**
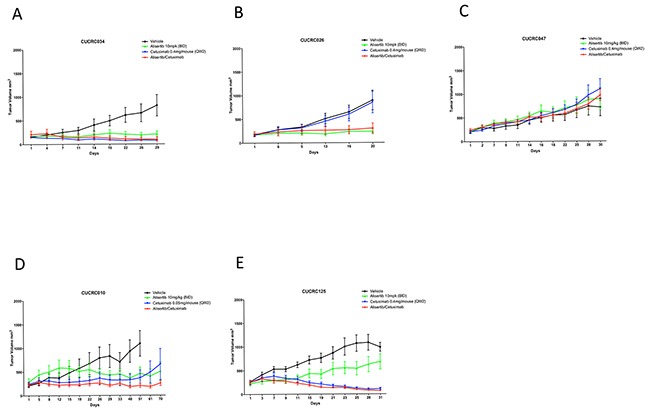
Growth curves of KRAS wildtype PDX models treated with alisertib, cetuximab, or the combinaiton: Five CRC PDX models were treated with vehicle, alisertib (10 mg/kg, QD) alone, cetuximab (0.4 ug/mouse, or 0.05 ug/mouse as designated, twice weekly) alone, or the combination for at least 20 days

**Figure 8:**
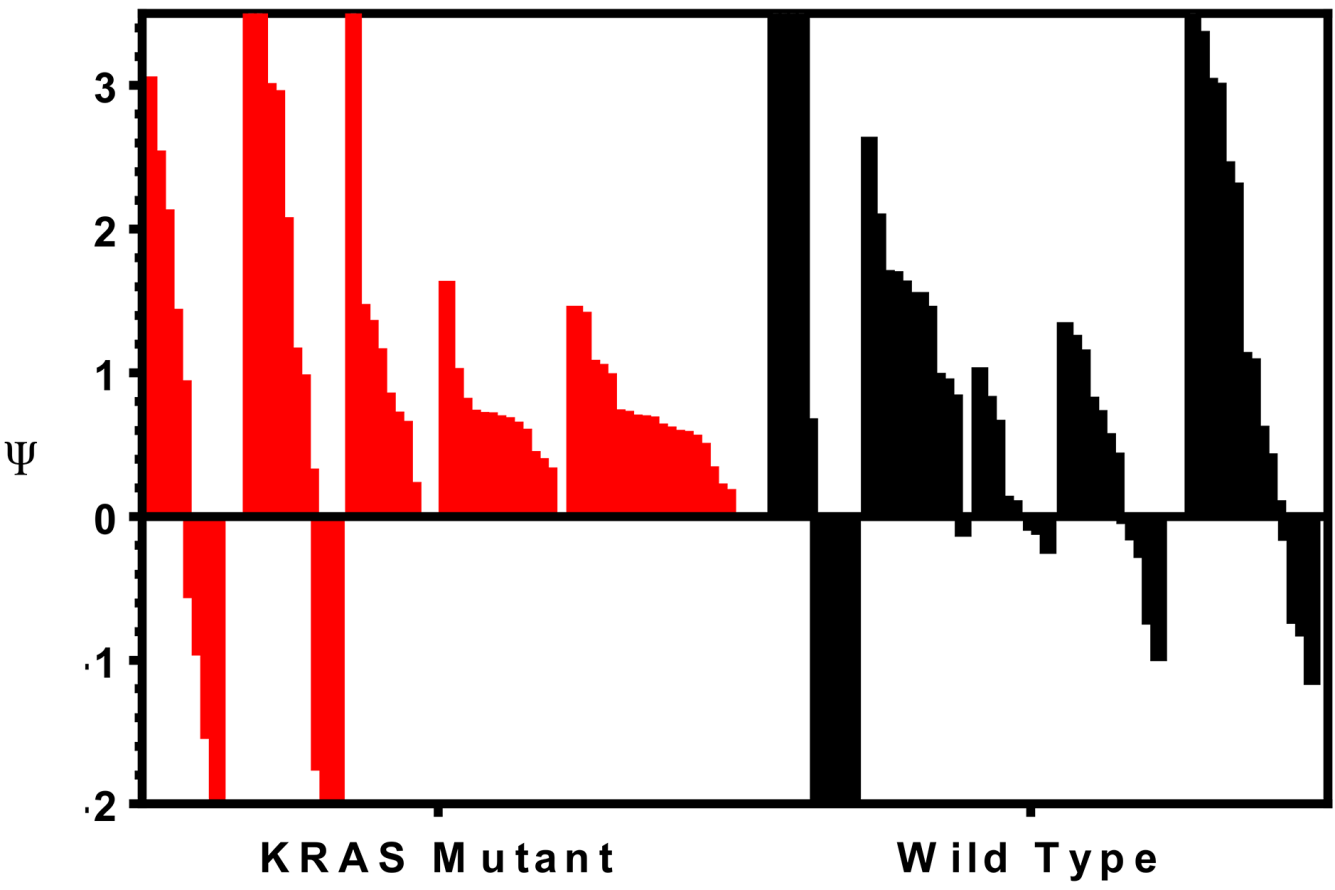
Assessment of the therapeutic benefit of adding irinotecan or cetuximab to alisertib Tumors were individually modeled in order to quantitatively assess the *in vivo* combinatorial effects of adding alisertib to standard of care agents. ψ is the term used in the mathematical model to define the degree of interaction. ψ values of 1 represent an additive effect, ψ > 1 represent a synergistic effect, 0 < ψ < 1 represent a less-than-additive effect, and ψ < 0 represent an antagonistic effect.

To determine if combination treatment with standard of care agents (irinotecan and cetuximab) was superior to single-agent alisertib, ten PDX models were treated with either single agent alisertib, irinotecan (KRAS^MT^ PDTX models) or cetuximab (KRAS^WT^ PDTX models) or the combination for at least 20 days. Cetuximab was used only for the KRAS^WT^, due to no benefit being observed clinically for KRAS^MT^ tumors treated with cetuximab. For the combination assessment, alisertib was administered at 10 mg/kg twice daily for both the single agent arm as well as in the combination group. As can be seen in Figure 6 (B, E) and 7 (D, E), four of the models show a modest combination effect when compared to the single agent arms. To further evaluate the effects of combination therapies, each tumor was individually modeled in order to quantitatively assess the *in vivo* combinatorial effects of adding alisertib to standard of care agents. In general, the addition of alisertib to standard of care treatments demonstrated improvement over single agent arms; however, the benefit was overall less than additive, but not antagonistic, as can be seen by ψ values being less than 1 but greater than 0 (Figure 8 and Supplementary Figure S1 and S2). ψ is the term used in the mathematical model to define the degree of interaction. ψ values of 1 represent an additive effect, ψ > 1 represent a synergistic effect, 0 < ψ < 1 represent a less-than-additive effect, and ψ < 0 represent an antagonistic effect.

The addition of alisertib to irinotecan in KRAS mutant PDX models demonstrated near additive to synergistic effects in 4 out 5 tumor types tested (CUCRC042 average ψ = 0.8, CUCRC098 average ψ = 0.7, CUCRC102 average ψ = 1.5 and CUCRC108 average ψ = 0.7) and a less than additive effect in one model (CUCRC007 average ψ = 0.5). In fact, out of 60 individual tumors modeled, the combination of alisertib to irinotecan was antagonistic (ψ < 0) in only 7 individual tumors, and additive to synergistic in 23 tumors (ψ ≥ 1). By contrast, the addition of alisertib to cetuximab therapy in KRAS wild-type PDTX models was far more variable with two models demonstrating synergy (CUCRC125 average ψ = 1.3 and CUCRC026 average ψ = 1.4), two models demonstrating a less than additive effect (CUCRC034 average ψ = 0.2 and CUCRC047 average ψ = 0.3) and one model exhibiting antagonism (CUCRC010 average ψ = −0.8). In the Kras wild-type models, a relatively even split between antagonism (18/57), less than additive (16/57) and additive to synergistic (23/57) was observed.

## DISCUSSION

Alisertib is an investigational, selective, small-molecule inhibitor of Aurora kinase A. As demonstrated with other Aurora kinase A inhibitors, alisertib has been shown to induce cell cycle arrest, polyploidy and cell death by mitotic catastrophe in a subset of tumor cell lines [18]. The current study was designed to evaluate the efficacy of alisertib in colon cancer models and to determine the antitumor effects of alisertib in combination with standard of care agents used to treat CRC. The results demonstrate that alisertib inhibits proliferation in a subset of CRC cell lines and induces cell cycle arrest, polyploidy, and apoptosis in the more sensitive lines. *In vivo*, this agent exhibits modest single agent activity against patient-derived CRC xenografts that was enhanced, to varying degrees, when combined with standard of care agents.

Previous preclinical studies have demonstrated that alisertib is effective at inhibiting proliferation of numerous tumor types including esophageal adenocarcinoma, pancreatic, and bladder cancer [17, 18, 21].

In addition to inhibiting proliferation in these models, induction of cell death and the cell cycle arrest was observed. The anti-tumor activity of alisertib exposure is not unexpected, but the results appear to be target dependent. For example, the pan-Aurora kinase inhibitors, PHA-739358 and PF-03814735 demonstrated inhibition of proliferation, induction of apoptosis and cell cycle arrest in multiple tumor types, including melanoma, HCC and SCLC [22–24]. As with the other tumor types, colorectal cancer cells treated with the Aurora kinase inhibitor BPR1K0609S1, displayed an increase in G2/M arrested cells and induction of apoptosis as demonstrated by an increase in the number of cells in sub-G1 of the cell cycle [25].

Other reports demonstrate that Aurora A may directly interact with p53 by phosphorylation, thus affecting its apoptotic activity [10]. For example, Aurora A inhibition with agents such as alisertib can lead to activation of p53 and its effectors. In some models, if p53 activity is lost, cells appear to be more sensitive to Aurora kinase A and B inhibition [26]. However in a triple negative breast cancer model, knockdown of wildtype p53 abrogated the alisertib apoptotic activity [15]. Assessment of p53 by immunoblotting in our studies demonstrated an induction of p53 in the sensitive p53^WT^ HCT116 CRC cell line at all time points, however after 48 hours of treatment with alisertib the induction is more pronounced. Conversely, in our p53 mutant model, we observed only a slight increase in p53 at 24 hours and no increase at 48 hours. No increase of p53 was observed in the two alisertib resistant CRC cell lines. These data are somewhat consistent with what has been observed previously in that p53 mutant cells do not upregulate p53 at early time points after exposure to alisertib [27]. This is also consistent with other tumor types, where p53^WT^ bladder cell lines demonstrated an increase in p53 following Aurora A inhibition for 48 hours [18]. p21, a known downstream target of p53 was also modestly increased following exposure to alisertib in the p53 wildtype cell lines whereas induction of p21 did not occur in the p53 mutant cell line which also exhibited only a minimal increase in p53. This is similar to what was observed by our group in a related study [27], however in that work we observed more apoptotic activity in the p53 mutant cell line whereas in the current study the induction of apoptosis was similar among the sensitive cell lines. The mechanism by which aurora kinase A enhances p53 degradation is not currently known. One model hypothesizes that aurora kinase A phosphorylates p53, thereby enhancing the binding of Mdm2 which then causes increased ubiquitination and degradation of p53 [10, 28]. By inhibiting aurora kinase A activity with alisertib, this degradation of p53 does not occur and p53 is able to induce apoptosis.

Consistent with previous data, a G2/M cell cycle arrest was observed in all cell lines tested. However following 48 hours of alisertib exposure, an greater increase in aneuploidy was observed in the HCT116 cells compared to the LS123 and COLO678 cells and may be due to the differential effects of Aurora B, which is associated with effects on mitotic slippage and aneuploidy [29]. At higher doses, alisertib inhibits Aurora B and the HCT116 cells may be more sensitive to this effect than the LS123 or COLO678 cell lines. This has been demonstrated in bladder cancer and upper gastrointestinal adenocarcinoma cell lines, where induction of polyploidy by alisertib is observed regardless of p53 mutational status [18, 30].

Alisertib has demonstrated efficacy in xenograft models of multiple tumor types, including bladder, multiple myeloma, neuroblastoma and esophageal adenocarcinoma [16–18, 31]. To better evaluate the clinical relevance of this compound in colorectal cancer, we utilized patient derived xenograft models (PDX) that more closely recapitulate the heterogeneity observed in patients. We treated twenty-one PDX models with alisertib and calculated the tumor growth inhibition index (TGII). Seven tumor models were classified as sensitive to single agent alisertib based on a TGII of < 20% with three models actually exhibiting regression.

In early Phase I clinical trials in patients with solid tumors, alisertib demonstrated a best response of stable disease of 7.3 months, and stable disease was durable for more than 6 months in 4 patients with colorectal cancer [19]. In addition, a Phase II study of alisertib in breast, head and neck, gastro-esophageal, small-cell and non-small-cell lung cancer demonstrated modest single agent activity, with hormone receptor and HER-2 positive breast cancer patients giving the best objective response [20]. With this modest activity in hand, and prior data reporting the ability of Aurora A inhibition to sensitize colorectal cancer cells to 5-fluorouracil, we elected to investigate the combinatorial effects of alisertib with standard of care therapies in our CRC PDX models [25]. In our PDX studies, alisertib appeared to only modestly improve the anti-tumor treatment effects of the standard therapies, irinotecan and cetuximab and while we did observe some tumor regression in a few of our models in combination, robust synergistic effects were not observed. Perhaps an explanation for these modest combinatorial effects is due to the fact that alisertib induces p21 and a G2/M cell cycle arrest and similarly it has been demonstrated that SN-38, the active metabolite of irinotecan, does not induce cell death in cells that are actively cell cycle-arrested [32]. This suggests that perhaps by sequencing the drugs (irinotecan → alisertib) enhancement of the combination effects can be achieved. These data suggest that such combinations with alisertib in CRC could be pursued if better patient selection and drug sequencing strategies are developed but also support the development of more rationally based hypothesis-driven combinations based on synthetic lethality or complementary cell cycle effects to achieve induction of robust apoptotic cell death [33].

## MATERIALS AND METHODS

### Chemicals and reagents

Alisertib was provided by Millennium Pharmaceuticals (Cambridge, MA). For *in vitro* work alisertib was dissolved in 100% DMSO at a concentration of 10 mmol/L. For *in vivo* studies, alisertib was dissolved in a half volume of 20% 2-hydroxypropyl-β-cyclodextrin in sterile water (w/v) and then diluted with a solution of 2% sodium bicarbonate in sterile water (w/v) to provide a final formulation in 10% 2-hydroxypropyl-β-cyclodextrin / 1% sodium bicarbonate.

### Cell lines, culture and proliferation

Human colorectal cancer cell lines were obtained from American Type Culture Collection, DSMZ, or the Korean Cell Line Bank. Geo cells, described previously [34], were kindly provided by Dr. Fortunato Ciardiello (Cattedra di Oncologia Medica, Dipartimento Medico-Chirurgico di Internistica Clinica e Sperimentale “F Magrassi e A Lanzara,” Seconda Universita' degli Studi di Napoli, Naples, Italy). KM12L4, KM12C, and KM20, described previously [35], were all kindly provided by Scott Kopetz (MD Anderson Cancer Center, Houston, TX). All cells were routinely cultured in RPMI 1640. All medium was supplemented with 10% FBS, 1% penicillin–streptomycin, and 1% MEM nonessential amino acids. All cells were kept at 37°C under an atmosphere containing 5% CO_2_. Cells were routinely tested for the presence of mycoplasma (MycoAlert; Cambrex BioScience). All CRC cell lines used in this study have been fully characterized and authenticated in the University of Colorado Cancer Center DNA Sequencing and Analysis Core.

Cells were seeded in 96-well black walled plates at 2000-8000 cells per well, depending on cell line growth kinetics. After 24h, alisertib was added at increasing concentrations and cells were incubated for an additional 72h. Proliferation was assessed by CyQuant assay according to the manufacturer's protocol (Life Technologies, Carlsbad, CA) and read on a Synergy 2 plate reader (Biotek, Winooski, VT).

### Immunoblotting

Cells were seeded in 6-well plates and allowed to attach for 24h. Cells were then incubated for 8, 12, 24h or 48h with varying concentrations of alisertib. Cells were then washed with PBS and lysed with RIPA buffer (Cell Signaling, Danvers, MA). After sonication and centrifugation, a total of 30 μg of protein lysate was loaded onto a NuPage gel (Life Technologies, Carlsbad, CA), electrophoresed, and transferred to a nitrocellulose membrane using the Pierce G2 FastBlotter (Thermo Fisher, Rockford, IL). The membrane was blocked and probed overnight with primary antibodies, washed for 10 minutes 3X with TBS/Tween 20, and probed with DyLight secondary antibodies (Cell signaling, Danvers, MA), and imaged using the Licor Odyssey (Licor, Lincoln, NE). All primary antibodies were purchased from Cell Signaling Technology (Danvers, MA) and diluted as per the manufactures' instructions.

### Apoptosis and cell cycle analysis

Cells were seeded in a 6-well plate and allowed to adhere for 24 hours. Alisertib was added at the indicated concentrations for 24 and 48 hours. Following exposure, cells were trypsinized, washed in PBS and stained with Annexin V and PI using the Dead Cell Apoptosis Kit (Life Technologies, Carlsbad, CA) and analyzed by flow cytometry. For cell cycle analysis, cells were plated and exposed to drug as described above. Cells were trypsinized, washed in PBS and resuspended in Krishan's stain and analyzed for cell cycle and ploidy using flow cytometry. Both analyses were performed by the University of Colorado Cancer Center Flow Cytometry Core.

### Patient-derived xenograft studies

Five to six week-old female athymic nude mice (Harlan Labs, Indianapolis, IN) were used for all animal studies, caged in groups of 5, kept on a 12 hour light/dark cycle, and given sterile food and water *ad libitum*. The PDX were generated as previously described [34]. Briefly, a tumor specimen was collected at the time of surgery from a consented patient at the University of Colorado Hospital. Tumor material remaining after histopathologic analysis was cut into 2 to 3 mm^3^ pieces, submerged in Matrigel, and implanted subcutaneously into the flank of five nude mice. After tumors were expanded through at least the F3 generation, they were excised, cut, and injected into the left and right flanks of mice for each xenograft study. When the average tumor size reached a volume of approximately 200 mm^3^, mice were randomized into the four different groups (vehicle, alisertib, irinotecan or cetuximab, and combination). Mice were monitored daily for signs of toxicity and weighed twice weekly. For single agent treatment alisertib was administered once daily at 30 mg/kg by oral gavage. For combination studies, alisertib was dosed at 10 mg/kg twice daily, irinotecan was dosed at 15 mg/kg weekly, and cetuximab was dosed at 0.4 mg/mouse twice weekly, except where noted differently. Tumor volume (equation for volume = (length x width^2^) x 0.52) was evaluated twice per week with digital calipers, using the Study Director software package (Studylog Systems, South San Francisco, CA). Tumor growth inhibition index was calculated from average volume of the treated (*V*_t_) and vehicle control (*V*_vc_) groups, with the equation: TGII = 100 × (*V*_t final_ -*V*_t initial_)/(*V*_vc final_ -*V*_vc intial_). TGII values of less than 20% were considered responsive, whereas TGII values of above 20% were considered nonresponsive. At the end of treatment, mice were sacrificed by CO_2_ overdose followed by cervical dislocation prior to removal of the tumors for further analysis.

### Data analysis

One-way ANOVA analyses with a Tukey post-test was used to determine statistical significance between multiple groups. Analyses were performed with Prism version 4.02. *P* values<0.05 were considered statistically significant.

Data from the combination *in vivo* study was modeled, similarly to a previously published model, to assess the therapeutic benefit of adding irinotecan or cetuximab to alisertib [36, 37]. All modeling was performed on individual animal tumor volume data and modeled with SAAM II version 2.3.1 (The Epsilon Group, Charlottesville, VA). Details of the model used can be found in the supplemental methods.

## SUPPLEMENTARY MATERIALS FIGURES





## References

[1] Carmena M, Earnshaw WC (2003). The cellular geography of aurora kinases. Nature reviews Molecular cell biology.

[2] Pitts TM, Davis SL, Eckhardt SG, Bradshaw-Pierce EL (2014). Targeting nuclear kinases in cancer: development of cell cycle kinase inhibitors. Pharmacology & therapeutics.

[3] Kollareddy M, Zheleva D, Dzubak P, Brahmkshatriya PS, Lepsik M, Hajduch M (2012). Aurora kinase inhibitors: progress towards the clinic. Investigational new drugs.

[4] Malumbres M, Perez de Castro I (2014). Aurora kinase A inhibitors: promising agents in antitumoral therapy. Expert opinion on therapeutic targets.

[5] Vader G, Lens SM (2008). The Aurora kinase family in cell division and cancer. Biochimica et biophysica acta.

[6] Anand S, Penrhyn-Lowe S, Venkitaraman AR (2003). AURORA-A amplification overrides the mitotic spindle assembly checkpoint, inducing resistance to Taxol. Cancer cell.

[7] Wu CC, Yu CT, Chang GC, Lai JM, Hsu SL (2011). Aurora-A promotes gefitinib resistance via a NF-kappaB signaling pathway in p53 knockdown lung cancer cells. Biochemical and biophysical research communications.

[8] Yang H, He L, Kruk P, Nicosia SV, Cheng JQ (2006). Aurora-A induces cell survival and chemoresistance by activation of Akt through a p53-dependent manner in ovarian cancer cells. Int J Cancer.

[9] Chen SS, Chang PC, Cheng YW, Tang FM, Lin YS (2002). Suppression of the STK15 oncogenic activity requires a transactivation-independent p53 function. The EMBO journal.

[10] Katayama H, Sasai K, Kawai H, Yuan ZM, Bondaruk J, Suzuki F, Fujii S, Arlinghaus RB, Czerniak BA, Sen S (2004). Phosphorylation by aurora kinase A induces Mdm2-mediated destabilization and inhibition of p53. Nature genetics.

[11] Mao JH, Wu D, Perez-Losada J, Jiang T, Li Q, Neve RM, Gray JW, Cai WW, Balmain A (2007). Crosstalk between Aurora-A and p53: frequent deletion or downregulation of Aurora-A in tumors from p53 null mice. Cancer cell.

[12] Meraldi P, Honda R, Nigg EA (2002). Aurora-A overexpression reveals tetraploidization as a major route to centrosome amplification in p53−/− cells. The EMBO journal.

[13] Ota T, Suto S, Katayama H, Han ZB, Suzuki F, Maeda M, Tanino M, Terada Y, Tatsuka M (2002). Increased mitotic phosphorylation of histone H3 attributable to AIM-1/Aurora-B overexpression contributes to chromosome number instability. Cancer research.

[14] Manfredi MG, Ecsedy JA, Chakravarty A, Silverman L, Zhang M, Hoar KM, Stroud SG, Chen W, Shinde V, Huck JJ, Wysong DR, Janowick DA, Hyer ML (2011). Characterization of Alisertib (MLN8237), an investigational small-molecule inhibitor of aurora A kinase using novel in vivo pharmacodynamic assays. Clinical cancer research.

[15] Tentler JJ, Ionkina AA, Tan AC, Newton TP, Pitts TM, Glogowska MJ, Kabos P, Sartorius CA, Sullivan KD, Espinosa JM, Eckhardt SG, Diamond JR (2015). p53 Family Members Regulate Phenotypic Response to Aurora Kinase A Inhibition in Triple-Negative Breast Cancer. Molecular cancer therapeutics.

[16] Gorgun G, Calabrese E, Hideshima T, Ecsedy J, Perrone G, Mani M, Ikeda H, Bianchi G, Hu Y, Cirstea D, Santo L, Tai YT, Nahar S (2010). A novel Aurora-A kinase inhibitor MLN8237 induces cytotoxicity and cell-cycle arrest in multiple myeloma. Blood.

[17] Sehdev V, Peng D, Soutto M, Washington MK, Revetta F, Ecsedy J, Zaika A, Rau TT, Schneider-Stock R, Belkhiri A, El-Rifai W (2012). The aurora kinase A inhibitor MLN8237 enhances cisplatin-induced cell death in esophageal adenocarcinoma cells. Molecular cancer therapeutics.

[18] Zhou N, Singh K, Mir MC, Parker Y, Lindner D, Dreicer R, Ecsedy JA, Zhang Z, Teh BT, Almasan A, Hansel DE (2013). The investigational Aurora kinase A inhibitor MLN8237 induces defects in cell viability and cell-cycle progression in malignant bladder cancer cells in vitro and in vivo. Clinical cancer research.

[19] Cervantes A, Elez E, Roda D, Ecsedy J, Macarulla T, Venkatakrishnan K, Rosello S, Andreu J, Jung J, Sanchis-Garcia JM, Piera A, Blasco I, Manos L (2012). Phase I pharmacokinetic/pharmacodynamic study of MLN8237, an investigational, oral, selective aurora a kinase inhibitor, in patients with advanced solid tumors. Clinical cancer research.

[20] Melichar B, Adenis A, Lockhart AC, Bennouna J, Dees EC, Kayaleh O, Obermannova R, DeMichele A, Zatloukal P, Zhang B, Ullmann CD, Schusterbauer C (2015). Safety and activity of alisertib, an investigational aurora kinase A inhibitor, in patients with breast cancer, small-cell lung cancer, non-small-cell lung cancer, head and neck squamous-cell carcinoma, and gastro-oesophageal adenocarcinoma: a five-arm phase 2 study. Lancet Oncol.

[21] Neel NF, Stratford JK, Shinde V, Ecsedy JA, Martin TD, Der CJ, Yeh JJ (2014). Response to MLN8237 in pancreatic cancer is not dependent on RalA phosphorylation. Molecular cancer therapeutics.

[22] Benten D, Keller G, Quaas A, Schrader J, Gontarewicz A, Balabanov S, Braig M, Wege H, Moll J, Lohse AW, Brummendorf TH (2009). Aurora kinase inhibitor PHA-739358 suppresses growth of hepatocellular carcinoma in vitro and in a xenograft mouse model. Neoplasia.

[23] Hook KE, Garza SJ, Lira ME, Ching KA, Lee NV, Cao J, Yuan J, Ye J, Ozeck M, Shi ST, Zheng X, Rejto PA, Kan JL, Christensen JG, Pavlicek A (2012). An integrated genomic approach to identify predictive biomarkers of response to the aurora kinase inhibitor PF-03814735. Molecular cancer therapeutics.

[24] Xie L, Meyskens FL (2013). The pan-Aurora kinase inhibitor, PHA-739358, induces apoptosis and inhibits migration in melanoma cell lines. Melanoma Res.

[25] Shionome Y, Lin WH, Shiao HY, Hsieh HP, Hsu JT, Ouchi T (2013). A novel aurora-A inhibitor, BPR1K0609S1, sensitizes colorectal tumor cells to 5-fluorofracil (5-FU) treatment. International journal of biological sciences.

[26] Marxer M, Ma HT, Man WY, Poon RY (2014). p53 deficiency enhances mitotic arrest and slippage induced by pharmacological inhibition of Aurora kinases. Oncogene.

[27] Davis SL, Robertson KM, Pitts TM, Tentler JJ, Bradshaw-Pierce EL, Klauck PJ, Bagby SM, Hyatt SL, Selby HM, Spreafico A, Ecsedy JA, Arcaroli JJ, Messersmith WA (2015). Combined inhibition of MEK and Aurora A kinase in KRAS/PIK3CA double-mutant colorectal cancer models. Front Pharmacol.

[28] Maki CG (1999). Oligomerization is required for p53 to be efficiently ubiquitinated by MDM2. J Biol Chem.

[29] Sorger PK, Dobles M, Tournebize R, Hyman AA (1997). Coupling cell division and cell death to microtubule dynamics. Curr Opin Cell Biol.

[30] Sehdev V, Katsha A, Ecsedy J, Zaika A, Belkhiri A, El-Rifai W (2013). The combination of alisertib, an investigational Aurora kinase A inhibitor, and docetaxel promotes cell death and reduces tumor growth in preclinical cell models of upper gastrointestinal adenocarcinomas. Cancer.

[31] Carol H, Boehm I, Reynolds CP, Kang MH, Maris JM, Morton CL, Gorlick R, Kolb EA, Keir ST, Wu J, Wozniak AE, Yang Y, Manfredi M (2011). Efficacy and pharmacokinetic/pharmacodynamic evaluation of the Aurora kinase A inhibitor MLN8237 against preclinical models of pediatric cancer. Cancer chemotherapy and pharmacology.

[32] Shah MA, Schwartz GK (2001). Cell cycle-mediated drug resistance: an emerging concept in cancer therapy. Clinical cancer research.

[33] Hilton JF, Shapiro GI (2014). Aurora kinase inhibition as an anticancer strategy. Journal of clinical oncology.

[34] Pitts TM, Tan AC, Kulikowski GN, Tentler JJ, Brown AM, Flanigan SA, Leong S, Coldren CD, Hirsch FR, Varella-Garcia M, Korch C, Eckhardt SG (2010). Development of an integrated genomic classifier for a novel agent in colorectal cancer: approach to individualized therapy in early development. Clinical cancer research.

[35] Morikawa K, Walker SM, Nakajima M, Pathak S, Jessup JM, Fidler IJ (1988). Influence of organ environment on the growth, selection, and metastasis of human colon carcinoma cells in nude mice. Cancer research.

[36] Koch G, Walz A, Lahu G, Schropp J (2009). Modeling of tumor growth and anticancer effects of combination therapy. Journal of pharmacokinetics and pharmacodynamics.

[37] Bradshaw-Pierce EL, Pitts TM, Kulikowski G, Selby H, Merz AL, Gustafson DL, Serkova NJ, Eckhardt SG, Weekes CD (2013). Utilization of quantitative in vivo pharmacology approaches to assess combination effects of everolimus and irinotecan in mouse xenograft models of colorectal cancer. PloS one.

