# Supplementary material for: Antitumor activity of the aurora a selective kinase inhibitor, alisertib, against preclinical models of colorectal cancer
Source: Oncotarget. 2016 Jul 1;7(31):50290–301. doi: 10.18632/oncotarget.10366 (PMC5226583; doi:10.18632/oncotarget.10366)
Supplement: Supplementary file 1 [file oncotarget-07-50290-s001.pdf]

Antitumor activity of the aurora a selective kinase inhibitor, alisertib, against preclinical models of colorectal cancer

SUPPLEMENTARY FIGURES

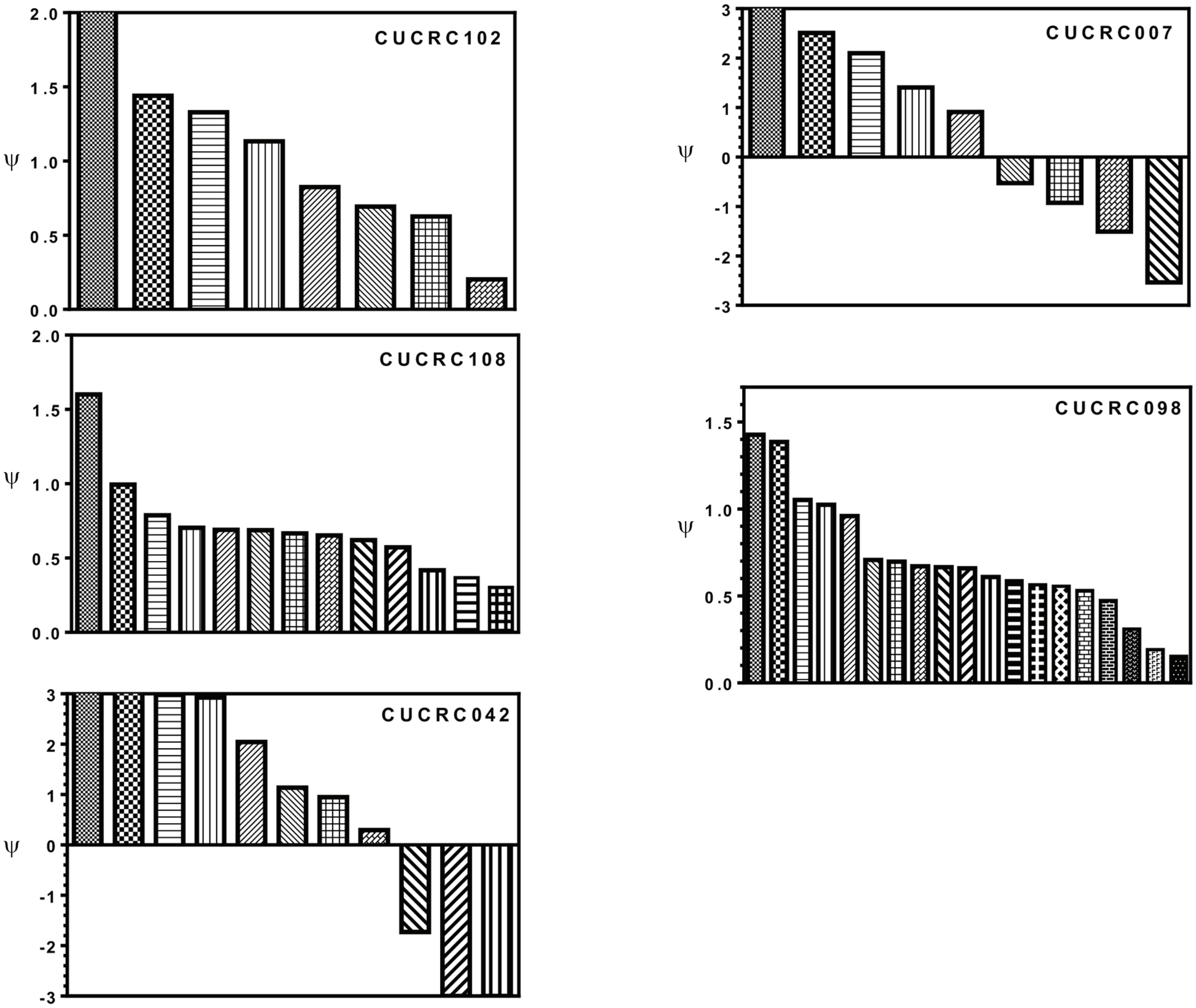

Supplementary Figure S1: Interaction term, ψ, for individual *KRAS* mutant tumors. Each bar represents an individual tumor.

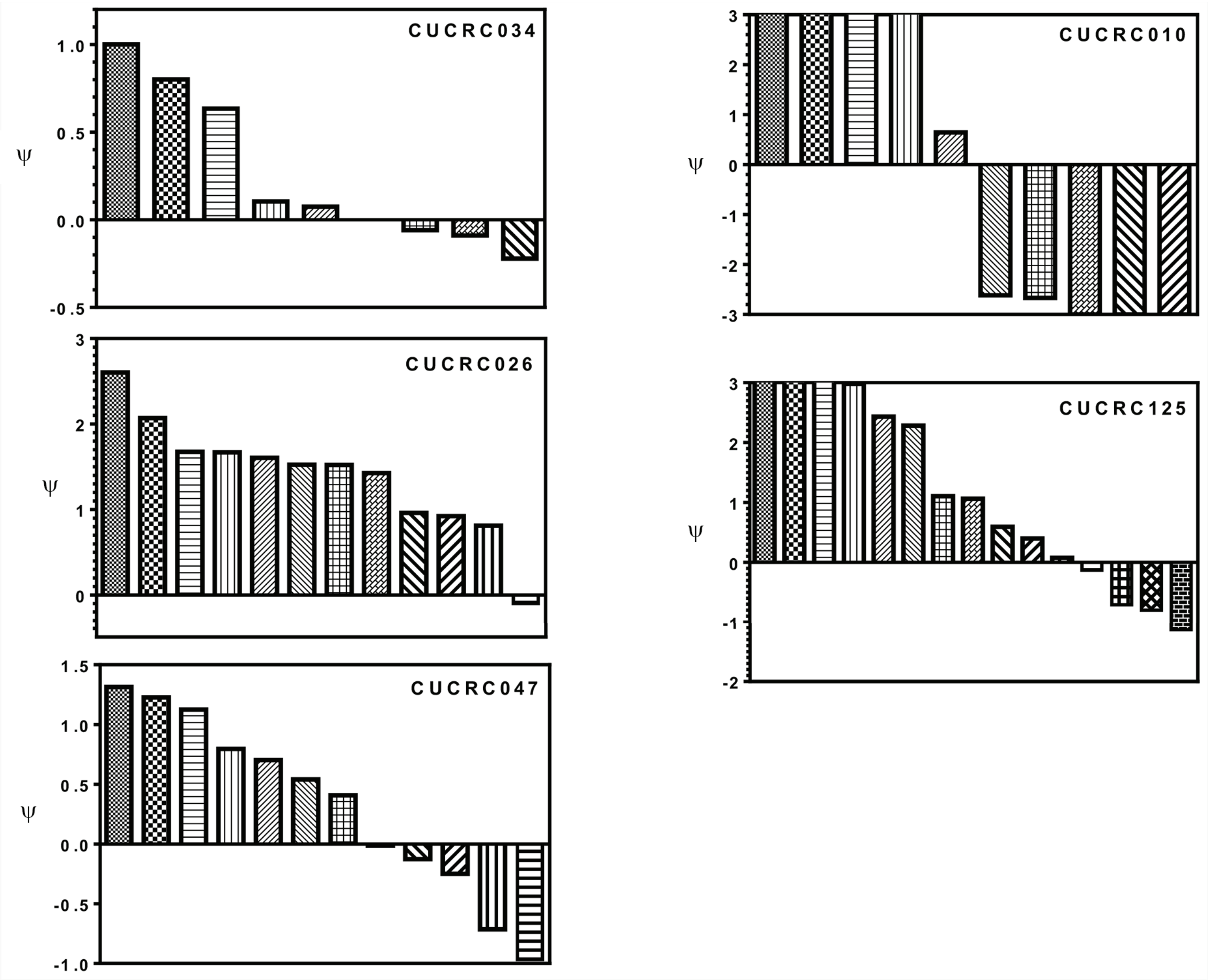

Supplementary Figure S2: Interaction term, ψ, for individual *KRAS* wild-type tumors. Each bar represents an individual tumor.
