# Supplementary material for: Antitumor activity of the aurora a selective kinase inhibitor, alisertib, against preclinical models of colorectal cancer
Source: Oncotarget. 2016 Jul 1;7(31):50290–301. doi: 10.18632/oncotarget.10366 (PMC5226583; doi:10.18632/oncotarget.10366)
Supplement: Supplementary file 2 [file oncotarget-07-50290-s002.docx]

Alisertib combination modeling

fits

### C U C R C 0 0 7 C o n t r o l

4 0 0

**T u m o r V o lu m e ( m m 3 )**

3 0 0

2 0 0

1 0 0

2 0 0 0


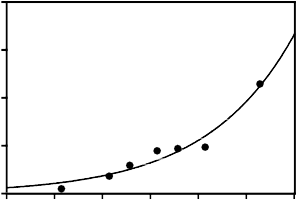


**T u m o r V o lu m e ( m m 3 )**

1 5 0 0

1 0 0 0

5 0 0

1 0 0 0


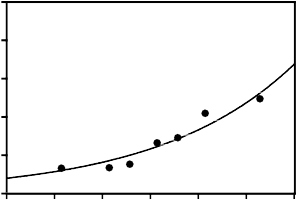


**T u m o r V o lu m e ( m m 3 )**

8 0 0

6 0 0

4 0 0

2 0 0

2 0 0 0


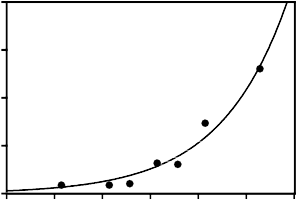


**T u m o r V o lu m e ( m m 3 )**

1 5 0 0

1 0 0 0

5 0 0

0

0 1 6 8 3 3 6 5 0 4 6 7 2 8 4 0 1 0 0 8

**T im e [ h ]**

0

0 1 6 8 3 3 6 5 0 4 6 7 2 8 4 0 1 0 0 8

**T im e [ h ]**

0

0 1 6 8 3 3 6 5 0 4 6 7 2 8 4 0 1 0 0 8

**T im e [ h ]**

0

0 1 6 8 3 3 6 5 0 4 6 7 2 8 4 0 1 0 0 8

**T im e [ h ]**

2 0 0 0

**T u m o r V o lu m e ( m m 3 )**

1 5 0 0

1 0 0 0

5 0 0

0


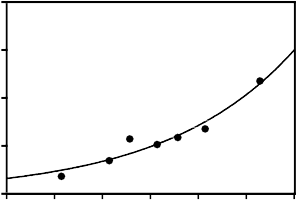


0 1 6 8 3 3 6 5 0 4 6 7 2 8 4 0 1 0 0 8

**T im e [ h ]**

4 0 0

**T u m o r V o lu m e ( m m 3 )**

3 0 0

2 0 0

1 0 0

0


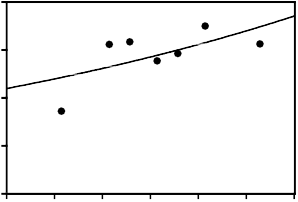


0 1 6 8 3 3 6 5 0 4 6 7 2 8 4 0 1 0 0 8

**T im e [ h ]**

3 0 0 0

**T u m o r V o lu m e ( m m 3 )**

2 0 0 0

1 0 0 0

0


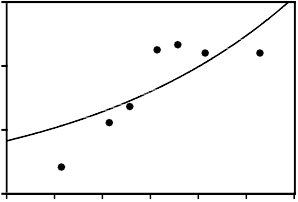


0 1 6 8 3 3 6 5 0 4 6 7 2 8 4 0 1 0 0 8

**T im e [ h ]**

3 0 0 0

**T u m o r V o lu m e ( m m 3 )**

2 0 0 0

1 0 0 0

0


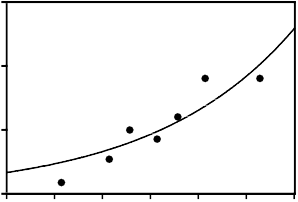


0 1 6 8 3 3 6 5 0 4 6 7 2 8 4 0 1 0 0 8

**T im e [ h ]**

1 0 0 0

**T u m o r V o lu m e ( m m 3 )**

8 0 0

6 0 0

4 0 0

2 0 0

0


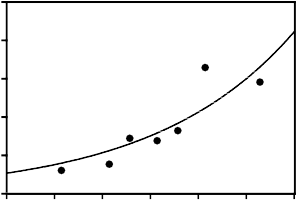


0 1 6 8 3 3 6 5 0 4 6 7 2 8 4 0 1 0 0 8

**T im e [ h ]**

1 0 0 0

**T u m o r V o lu m e ( m m 3 )**

8 0 0

6 0 0

4 0 0

2 0 0

0


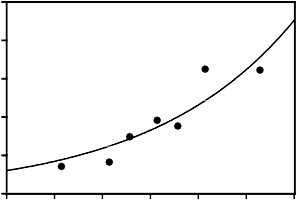


0 1 6 8 3 3 6 5 0 4 6 7 2 8 4 0 1 0 0 8

**T im e [ h ]**

**C U C R C 0 0 7 Ir in o te c a n**

4 0 0

**T u m o r V o lu m e ( m m 3 )**

3 0 0

2 0 0

1 0 0

0


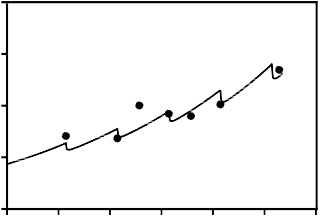


0 1 6 8 3 3 6 5 0 4 6 7 2 8 4 0 1 0 0 8

**T im e [ h ]**

8 0 0

**T u m o r V o lu m e ( m m 3 )**

6 0 0

4 0 0

2 0 0

0


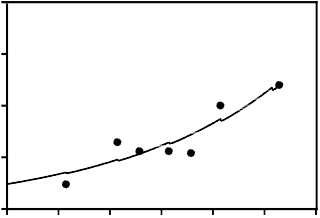


0 1 6 8 3 3 6 5 0 4 6 7 2 8 4 0 1 0 0 8

**T im e [ h ]**

1 0 0 0

**T u m o r V o lu m e ( m m 3 )**

8 0 0

6 0 0

4 0 0

2 0 0

0


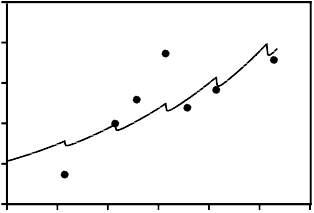


0 1 6 8 3 3 6 5 0 4 6 7 2 8 4 0 1 0 0 8

**T im e [ h ]**

8 0 0

**T u m o r V o lu m e ( m m 3 )**

6 0 0

4 0 0

2 0 0

0


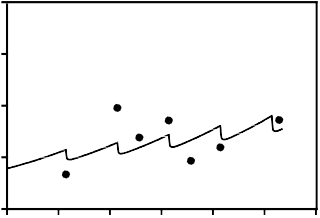


0 1 6 8 3 3 6 5 0 4 6 7 2 8 4 0 1 0 0 8

**T im e [ h ]**

4 0 0

**T u m o r V o lu m e ( m m 3 )**

3 0 0

2 0 0

1 0 0

0


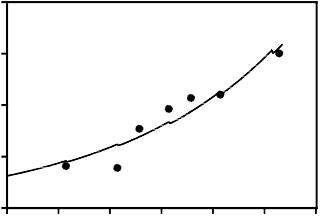


0 1 6 8 3 3 6 5 0 4 6 7 2 8 4 0 1 0 0 8

**T im e [ h ]**

4 0 0

**T u m o r V o lu m e ( m m 3 )**

3 0 0

2 0 0

1 0 0

0


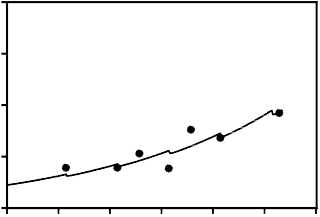


0 1 6 8 3 3 6 5 0 4 6 7 2 8 4 0 1 0 0 8

**T im e [ h ]**

1 0 0 0

**T u m o r V o lu m e ( m m 3 )**

8 0 0

6 0 0

4 0 0

2 0 0

0


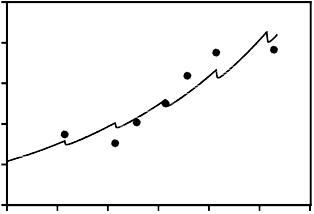


0 1 6 8 3 3 6 5 0 4 6 7 2 8 4 0 1 0 0 8

**T im e [ h ]**

4 0 0

**T u m o r V o lu m e ( m m 3 )**

2 0 0

0


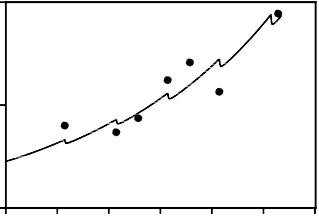


0 1 6 8 3 3 6 5 0 4 6 7 2 8 4 0 1 0 0 8

**T im e [ h ]**

8 0 0

**T u m o r V o lu m e ( m m 3 )**

6 0 0

4 0 0

2 0 0

0


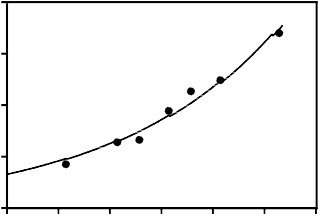


0 1 6 8 3 3 6 5 0 4 6 7 2 8 4 0 1 0 0 8

**T im e [ h ]**

2 0 0 0

**T u m o r V o lu m e ( m m 3 )**

1 5 0 0

1 0 0 0

5 0 0

0


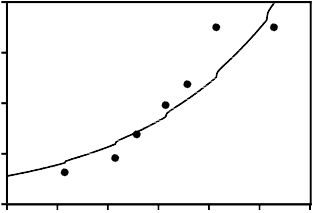


0 1 6 8 3 3 6 5 0 4 6 7 2 8 4 0 1 0 0 8

**T im e [ h ]**

8 0 0

**T u m o r V o lu m e ( m m 3 )**

6 0 0

4 0 0

2 0 0

0


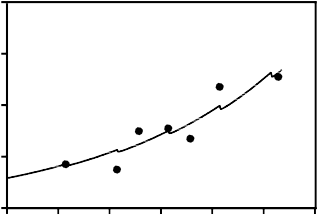


0 1 6 8 3 3 6 5 0 4 6 7 2 8 4 0 1 0 0 8

**T im e [ h ]**

4 0 0

**T u m o r V o lu m e ( m m 3 )**

3 0 0

2 0 0

1 0 0

0


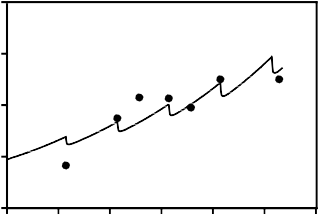


0 1 6 8 3 3 6 5 0 4 6 7 2 8 4 0 1 0 0 8

**T im e [ h ]**

**C U C R C 0 0 7 A l is e r t ib**

2 5 0 0

**T u m o r V o lu m e ( m m 3 )**

2 0 0 0

1 5 0 0

1 0 0 0

5 0 0

0


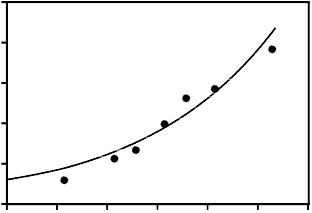


0 1 6 8 3 3 6 5 0 4 6 7 2 8 4 0 1 0 0 8

**T im e [ h ]**

4 0 0

**T u m o r V o lu m e ( m m 3 )**

3 0 0

2 0 0

1 0 0

0


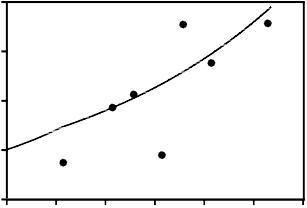


0 1 6 8 3 3 6 5 0 4 6 7 2 8 4 0 1 0 0 8

**T im e [ h ]**

4 0 0

**T u m o r V o lu m e ( m m 3 )**

3 0 0

2 0 0

1 0 0

0


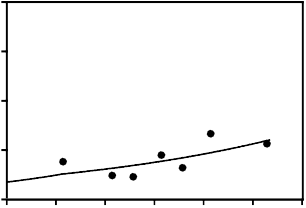


0 1 6 8 3 3 6 5 0 4 6 7 2 8 4 0 1 0 0 8

**T im e [ h ]**

1 5 0 0

**T u m o r V o lu m e ( m m 3 )**

1 0 0 0

5 0 0

0


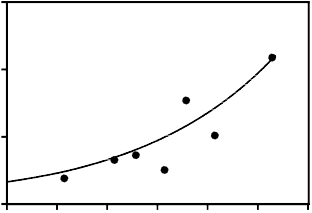


0 1 6 8 3 3 6 5 0 4 6 7 2 8 4 0 1 0 0 8

**T im e [ h ]**

4 0 0

**T u m o r V o lu m e ( m m 3 )**

2 0 0

0


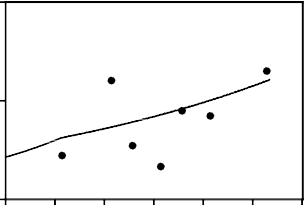


0 1 6 8 3 3 6 5 0 4 6 7 2 8 4 0 1 0 0 8

**T im e [ h ]**

3 0 0 0

**T u m o r V o lu m e ( m m 3 )**

2 0 0 0

1 0 0 0

0


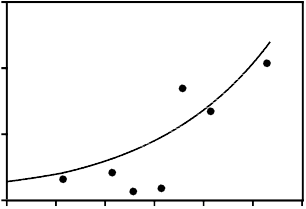


0 1 6 8 3 3 6 5 0 4 6 7 2 8 4 0 1 0 0 8

**T im e [ h ]**

4 0 0

**T u m o r V o lu m e ( m m 3 )**

2 0 0

0


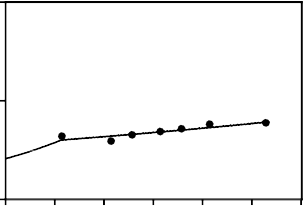


0 1 6 8 3 3 6 5 0 4 6 7 2 8 4 0 1 0 0 8

**T im e [ h ]**

1 0 0 0

**T u m o r V o lu m e ( m m 3 )**

8 0 0

6 0 0

4 0 0

2 0 0

0


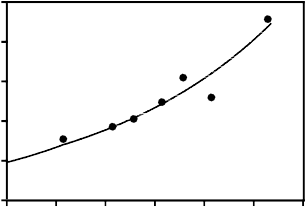


0 1 6 8 3 3 6 5 0 4 6 7 2 8 4 0 1 0 0 8

**T im e [ h ]**

4 0 0

**T u m o r V o lu m e ( m m 3 )**

3 0 0

2 0 0

1 0 0

0


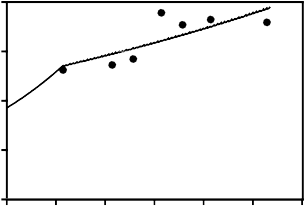


0 1 6 8 3 3 6 5 0 4 6 7 2 8 4 0 1 0 0 8

**T im e [ h ]**

**C U C R C 0 0 7 C o m b in a t io n**

**4 0 0**

**T u m o r V o lu m e ( m m 3 )**

**3 0 0**

**2 0 0**

**1 0 0**

**0**


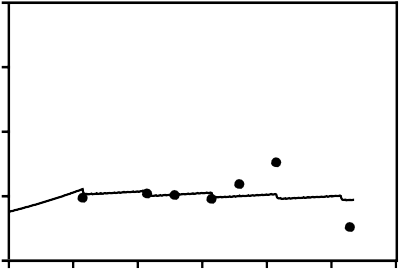


**0 1 6 8 3 3 6 5 0 4 6 7 2 8 4 0 1 0 0 8**

**T im e [ h ]**

**2 5 0 0**

**T u m o r V o lu m e ( m m 3 )**

**2 0 0 0**

**1 5 0 0**

**1 0 0 0**

**5 0 0**

**0**


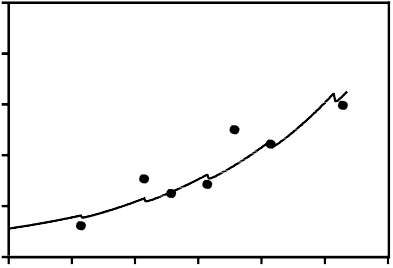


**0 1 6 8 3 3 6 5 0 4 6 7 2 8 4 0 1 0 0 8**

**T im e [ h ]**

**8 0 0**

**T u m o r V o lu m e ( m m 3 )**

**6 0 0**

**4 0 0**

**2 0 0**

**0**


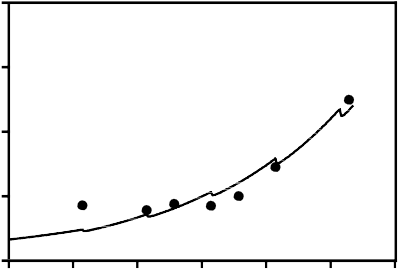


**0 1 6 8 3 3 6 5 0 4 6 7 2 8 4 0 1 0 0 8**

**T im e [ h ]**

**1 5 0 0**

**T u m o r V o lu m e ( m m 3 )**

**1 0 0 0**

**5 0 0**

**0**


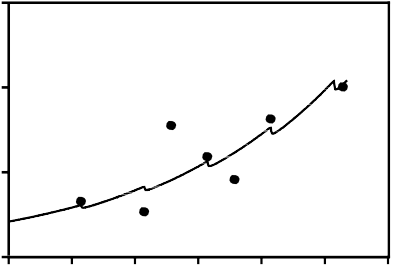


**0 1 6 8 3 3 6 5 0 4 6 7 2 8 4 0 1 0 0 8**

**T im e [ h ]**

**4 0 0**

**T u m o r V o lu m e ( m m 3 )**

**3 0 0**

**2 0 0**

**1 0 0**

**0**


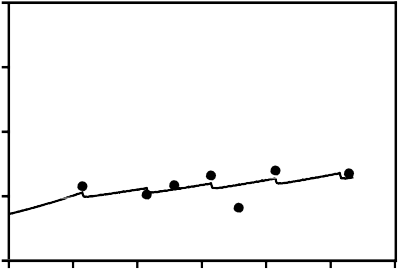


**0 1 6 8 3 3 6 5 0 4 6 7 2 8 4 0 1 0 0 8**

**T im e [ h ]**

**4 0 0**

**T u m o r V o lu m e ( m m 3 )**

**3 0 0**

**2 0 0**

**1 0 0**

**0**


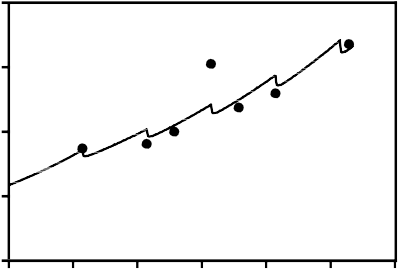


**0 1 6 8 3 3 6 5 0 4 6 7 2 8 4 0 1 0 0 8**

**T im e [ h ]**

**4 0 0**

**T u m o r V o lu m e ( m m 3 )**

**3 0 0**

**2 0 0**

**1 0 0**

**0**


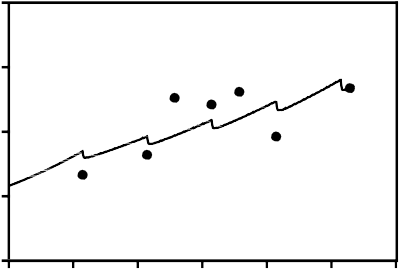


**0 1 6 8 3 3 6 5 0 4 6 7 2 8 4 0 1 0 0 8**

**T im e [ h ]**

**4 0 0**

**T u m o r V o lu m e ( m m 3 )**

**3 0 0**

**2 0 0**

**1 0 0**

**0**


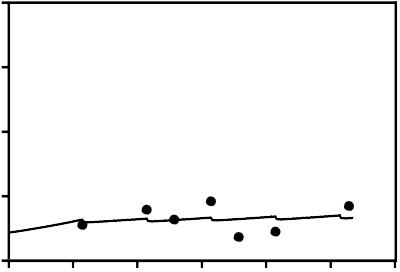


**0 1 6 8 3 3 6 5 0 4 6 7 2 8 4 0 1 0 0 8**

**T im e [ h ]**

**2 5 0 0**

**T u m o r V o lu m e ( m m 3 )**

**2 0 0 0**

**1 5 0 0**

**1 0 0 0**

**5 0 0**

**0**


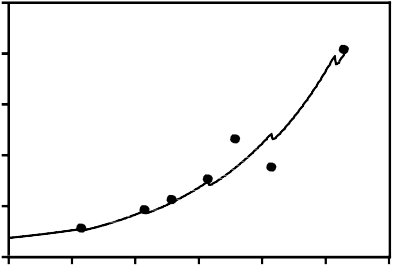


**0 1 6 8 3 3 6 5 0 4 6 7 2 8 4 0 1 0 0 8**

**T im e [ h ]**

**C U C R C 0 4 2 C o n t r o l**

**6 0 0**

T u m o r V o lu m e ( m m 3 )

**4 0 0**

**2 0 0**

**0**


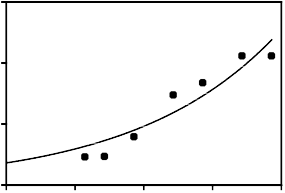


**0 1 6 8 3 3 6 5 0 4 6 7 2**

T im e [ h ]

2 0 0 0

T u m o r V o lu m e ( m m 3 )

1 0 0 0

0


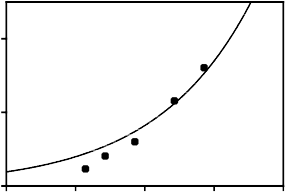


0 1 6 8 3 3 6 5 0 4 6 7 2

T im e [ h ]

8 0 0

T u m o r V o lu m e ( m m 3 )

6 0 0

4 0 0

2 0 0

0


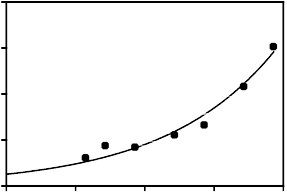


0 1 6 8 3 3 6 5 0 4 6 7 2

T im e [ h ]

2 0 0 0

T u m o r V o lu m e ( m m 3 )

1 0 0 0

0


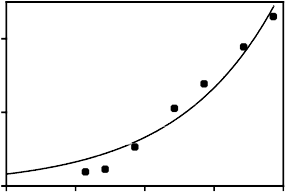


0 1 6 8 3 3 6 5 0 4 6 7 2

T im e [ h ]

1 5 0 0

T u m o r V o lu m e ( m m 3 )

1 0 0 0

5 0 0

0


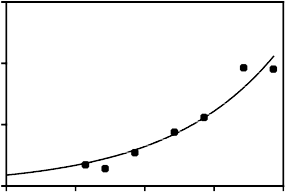


0 1 6 8 3 3 6 5 0 4 6 7 2

T im e [ h ]

5 0 0

T u m o r V o lu m e ( m m 3 )

4 0 0

3 0 0

2 0 0

1 0 0

0


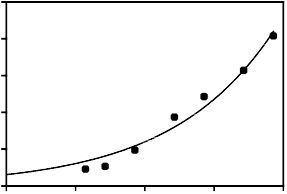


0 1 6 8 3 3 6 5 0 4 6 7 2

T im e [ h ]

1 5 0 0

T u m o r V o lu m e ( m m 3 )

1 0 0 0

5 0 0

0


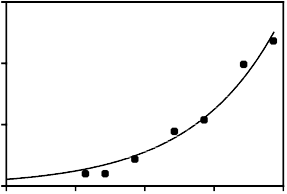


0 1 6 8 3 3 6 5 0 4 6 7 2

T im e [ h ]

8 0 0

T u m o r V o lu m e ( m m 3 )

6 0 0

4 0 0

2 0 0

0


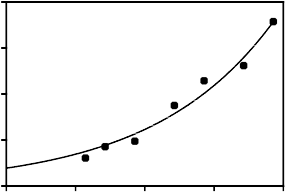


0 1 6 8 3 3 6 5 0 4 6 7 2

T im e [ h ]

2 5 0

T u m o r V o lu m e ( m m 3 )

2 0 0

1 5 0

1 0 0

5 0

0


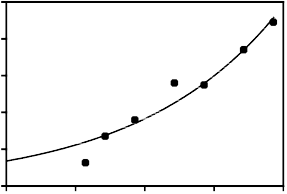


0 1 6 8 3 3 6 5 0 4 6 7 2

T im e [ h ]

1 0 0 0

T u m o r V o lu m e ( m m 3 )

5 0 0

0


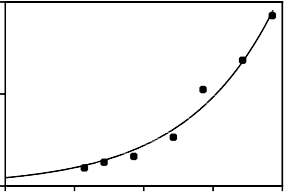


0 1 6 8 3 3 6 5 0 4 6 7 2

T im e [ h ]

**C U C R C 0 4 2 Ir in o te c a n**

1 5 0 0

**T u m o r V o lu m e ( m m 3 )**

**1 0 0 0**

**5 0 0**

**0**


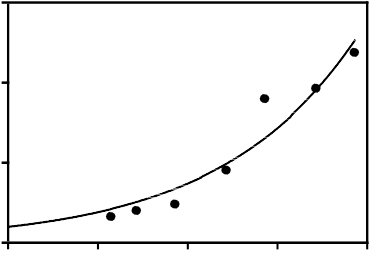


**0 1 6 8 3 3 6 5 0 4 6 7 2**

**T im e [ h ]**

**8 0 0**

**T u m o r V o lu m e ( m m 3 )**

**6 0 0**

**4 0 0**

**2 0 0**

**0**


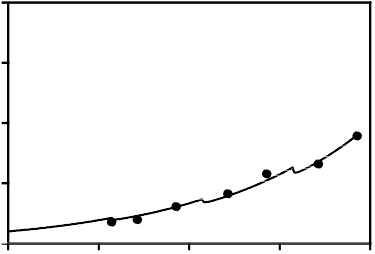


**0 1 6 8 3 3 6 5 0 4 6 7 2**

**T im e [ h ]**

**2 0 0 0**

**T u m o r V o lu m e ( m m 3 )**

**1 5 0 0**

**1 0 0 0**

**5 0 0**

**0**


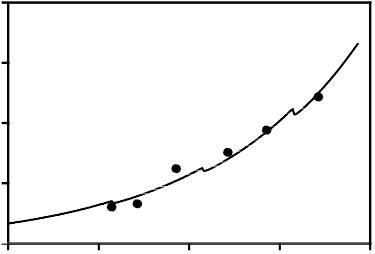


**0 1 6 8 3 3 6 5 0 4 6 7 2**

**T im e [ h ]**

**2 5 0 0**

**T u m o r V o lu m e ( m m 3 )**

**2 0 0 0**

**1 5 0 0**

**1 0 0 0**

**5 0 0**

**0**


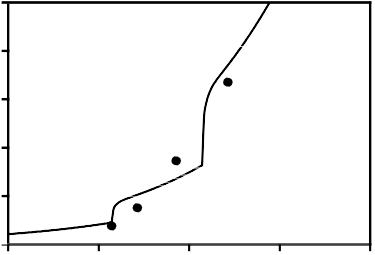


**0 1 6 8 3 3 6 5 0 4 6 7 2**

**T im e [ h ]**

**4 0 0**

**T u m o r V o lu m e ( m m 3 )**

**3 0 0**

**2 0 0**

**1 0 0**

**0**


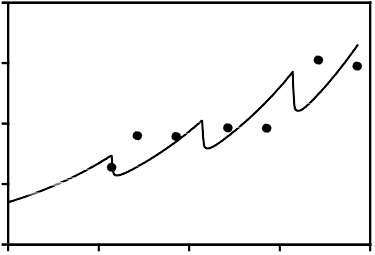


**0 1 6 8 3 3 6 5 0 4 6 7 2**

**T im e [ h ]**

**8 0 0**

**T u m o r V o lu m e ( m m 3 )**

**6 0 0**

**4 0 0**

**2 0 0**

**0**


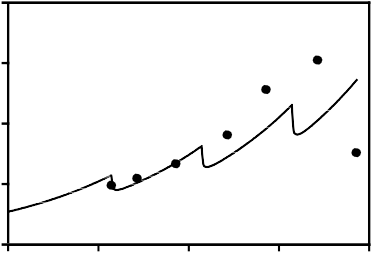


**0 1 6 8 3 3 6 5 0 4 6 7 2**

**T im e [ h ]**

**8 0 0**

**T u m o r V o lu m e ( m m 3 )**

**6 0 0**

**4 0 0**

**2 0 0**

**0**


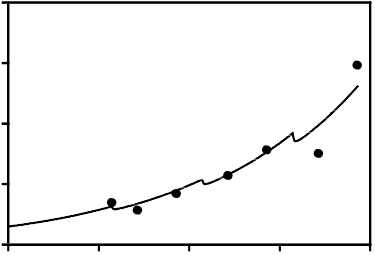


**0 1 6 8 3 3 6 5 0 4 6 7 2**

**T im e [ h ]**

**3 0 0 0**

**T u m o r V o lu m e ( m m 3 )**

**2 0 0 0**

**1 0 0 0**

**0**


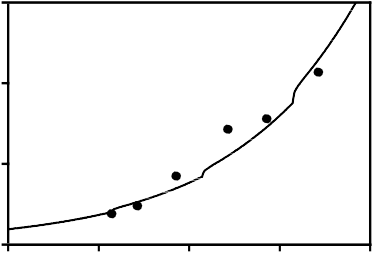


**0 1 6 8 3 3 6 5 0 4 6 7 2**

**T im e [ h ]**

**1 5 0 0**

**T u m o r V o lu m e ( m m 3 )**

**1 0 0 0**

**5 0 0**

**0**


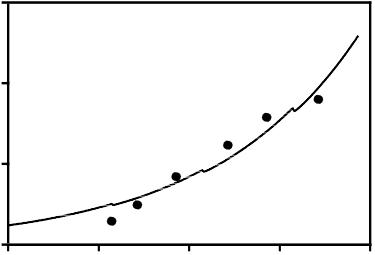


**0 1 6 8 3 3 6 5 0 4 6 7 2**

**T im e [ h ]**

**C U C R C 0 4 2 A l is e r t ib**

**8 0 0**

**T u m o r V o lu m e ( m m 3 )**

**6 0 0**

**4 0 0**

**2 0 0**

**0**


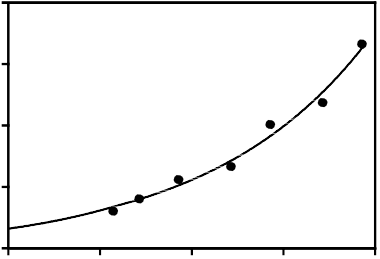


**0 1 6 8 3 3 6 5 0 4 6 7 2**

**T im e [ h ]**

**8 0 0**

**T u m o r V o lu m e ( m m 3 )**

**6 0 0**

**4 0 0**

**2 0 0**

**0**


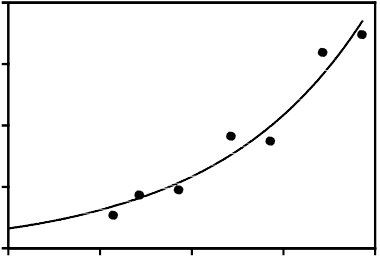


**0 1 6 8 3 3 6 5 0 4 6 7 2**

**T im e [ h ]**

**1 0 0 0**

**T u m o r V o lu m e ( m m 3 )**

**8 0 0**

**6 0 0**

**4 0 0**

**2 0 0**

**0**


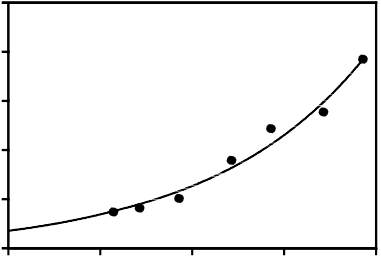


**0 1 6 8 3 3 6 5 0 4 6 7 2**

**T im e [ h ]**

**1 0 0 0**

**T u m o r V o lu m e ( m m 3 )**

**8 0 0**

**6 0 0**

**4 0 0**

**2 0 0**

**0**


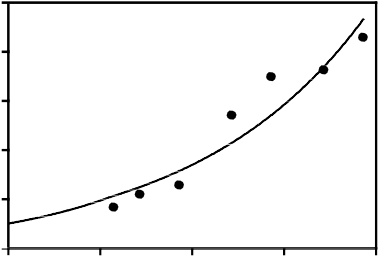


**0 1 6 8 3 3 6 5 0 4 6 7 2**

**T im e [ h ]**

**5 0 0**

**T u m o r V o lu m e ( m m 3 )**

**4 0 0**

**3 0 0**

**2 0 0**

**1 0 0**

**0**


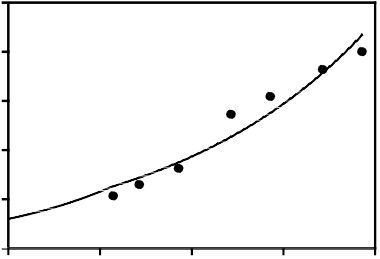


**0 1 6 8 3 3 6 5 0 4 6 7 2**

**T im e [ h ]**

**3 0 0**

**T u m o r V o lu m e ( m m 3 )**

**2 0 0**

**1 0 0**

**0**


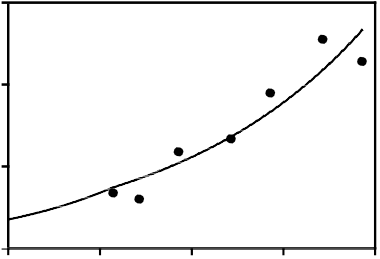


**0 1 6 8 3 3 6 5 0 4 6 7 2**

**T im e [ h ]**

**1 5 0 0**

**T u m o r V o lu m e ( m m 3 )**

**1 0 0 0**

**5 0 0**

**0**


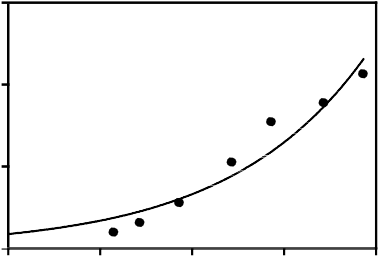


**0 1 6 8 3 3 6 5 0 4 6 7 2**

**T im e [ h ]**

**C U C R C 0 4 2 C o m b in a t io n**

**2 5 0**

T u m o r V o lu m e ( m m 3 )

**2 0 0**

**1 5 0**

**1 0 0**

**5 0**

**0**


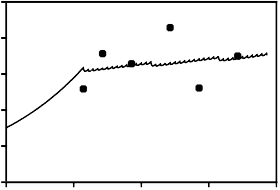


**0 1 6 8 3 3 6 5 0 4 6 7 2**

T im e [ h ]

**3 0 0 0**

T u m o r V o lu m e ( m m 3 )

**2 0 0 0**

**1 0 0 0**

**0**


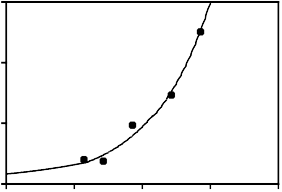


**0 1 6 8 3 3 6 5 0 4 6 7 2**

T im e [ h ]

**2 5 0 0**

T u m o r V o lu m e ( m m 3 )

**2 0 0 0**

**1 5 0 0**

**1 0 0 0**

**5 0 0**

**0**


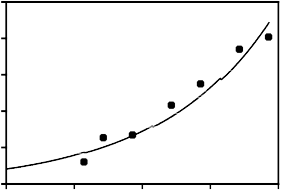


**0 1 6 8 3 3 6 5 0 4 6 7 2**

T im e [ h ]

**2 0 0 0**

T u m o r V o lu m e ( m m 3 )

**1 5 0 0**

**1 0 0 0**

**5 0 0**

**0**


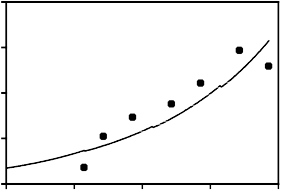


**0 1 6 8 3 3 6 5 0 4 6 7 2**

T im e [ h ]

**8 0 0**

T u m o r V o lu m e ( m m 3 )

**6 0 0**

**4 0 0**

**2 0 0**

**0**


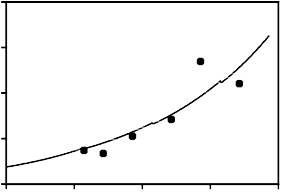


**0 1 6 8 3 3 6 5 0 4 6 7 2**

T im e [ h ]

**2 5 0 0**

T u m o r V o lu m e ( m m 3 )

**2 0 0 0**

**1 5 0 0**

**1 0 0 0**

**5 0 0**

**0**


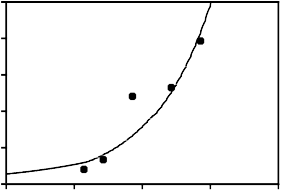


**0 1 6 8 3 3 6 5 0 4 6 7 2**

T im e [ h ]

**1 0 0 0**

T u m o r V o lu m e ( m m 3 )

**8 0 0**

**6 0 0**

**4 0 0**

**2 0 0**

**0**


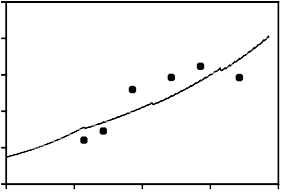


**0 1 6 8 3 3 6 5 0 4 6 7 2**

T im e [ h ]

**1 0 0 0**

T u m o r V o lu m e ( m m 3 )

**8 0 0**

**6 0 0**

**4 0 0**

**2 0 0**

**0**


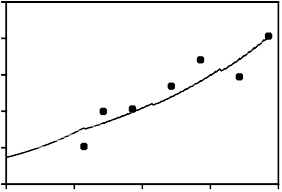


**0 1 6 8 3 3 6 5 0 4 6 7 2**

T im e [ h ]

**6 0 0**

T u m o r V o lu m e ( m m 3 )

**4 0 0**

**2 0 0**

**0**


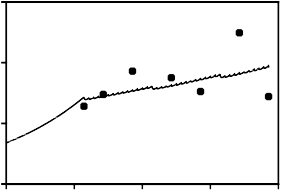


**0 1 6 8 3 3 6 5 0 4 6 7 2**

T im e [ h ]

**6 0 0**

T u m o r V o lu m e ( m m 3 )

**4 0 0**

**2 0 0**

**0**


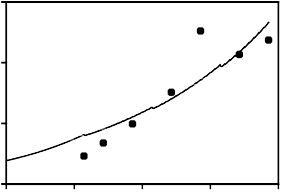


**0 1 6 8 3 3 6 5 0 4 6 7 2**

T im e [ h ]

**2 5 0 0**

T u m o r V o lu m e ( m m 3 )

**2 0 0 0**

**1 5 0 0**

**1 0 0 0**

**5 0 0**

**0**


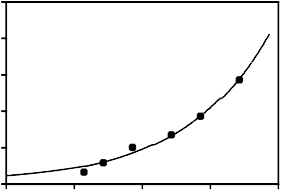


**0 1 6 8 3 3 6 5 0 4 6 7 2**

T im e [ h ]

**C U C R C 0 9 8 C o n t r o l**

2 0 0 0

T u m o r V o lu m e ( m m 3 )

1 5 0 0

1 0 0 0

5 0 0

0


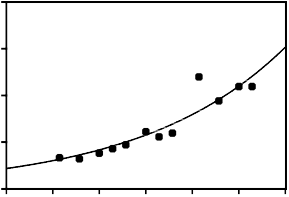


0 1 6 8 3 3 6 5 0 4 6 7 2 8 4 0 1 0 0 8

T im e [ h ]

**3 0 0 0**

T u m o r V o lu m e ( m m 3 )

**2 0 0 0**

**1 0 0 0**

**0**


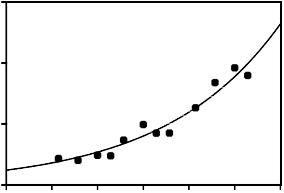


**0 1 6 8 3 3 6 5 0 4 6 7 2 8 4 0 1 0 0 8**

T im e [ h ]

**1 5 0 0**

T u m o r V o lu m e ( m m 3 )

**1 0 0 0**

**5 0 0**

**0**


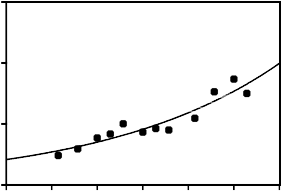


**0 1 6 8 3 3 6 5 0 4 6 7 2 8 4 0 1 0 0 8**

T im e [ h ]

2 5 0 0

T u m o r V o lu m e ( m m 3 )

2 0 0 0

1 5 0 0

1 0 0 0

5 0 0

0


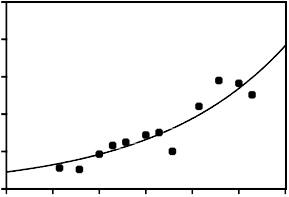


0 1 6 8 3 3 6 5 0 4 6 7 2 8 4 0 1 0 0 8

T im e [ h ]

**1 5 0 0**

T u m o r V o lu m e ( m m 3 )

**1 0 0 0**

**5 0 0**

**0**


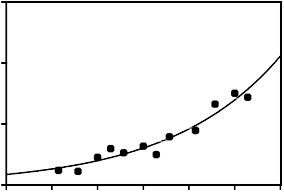


**0 1 6 8 3 3 6 5 0 4 6 7 2 8 4 0 1 0 0 8**

T im e [ h ]

**1 0 0 0 0**

T u m o r V o lu m e ( m m 3 )

**8 0 0 0**

**6 0 0 0**

**4 0 0 0**

**2 0 0 0**

**0**


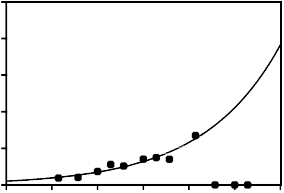


**0 1 6 8 3 3 6 5 0 4 6 7 2 8 4 0 1 0 0 8**

T im e [ h ]

3 0 0 0

T u m o r V o lu m e ( m m 3 )

2 0 0 0

1 0 0 0

0


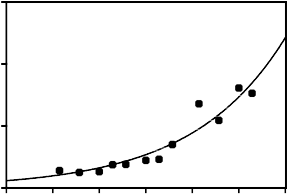


0 1 6 8 3 3 6 5 0 4 6 7 2 8 4 0 1 0 0 8

T im e [ h ]

**4 0 0 0**

T u m o r V o lu m e ( m m 3 )

**3 0 0 0**

**2 0 0 0**

**1 0 0 0**

**0**


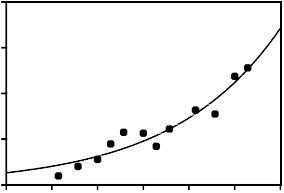


**0 1 6 8 3 3 6 5 0 4 6 7 2 8 4 0 1 0 0 8**

T im e [ h ]

1 5 0 0

T u m o r V o lu m e ( m m 3 )

1 0 0 0

5 0 0

0


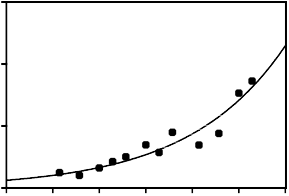


0 1 6 8 3 3 6 5 0 4 6 7 2 8 4 0 1 0 0 8

T im e [ h ]

6 0 0 0

T u m o r V o lu m e ( m m 3 )

4 0 0 0

2 0 0 0

0


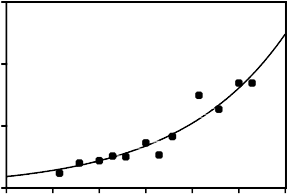


0 1 6 8 3 3 6 5 0 4 6 7 2 8 4 0 1 0 0 8

T im e [ h ]

5 0 0

T u m o r V o lu m e ( m m 3 )

4 0 0

3 0 0

2 0 0

1 0 0

0


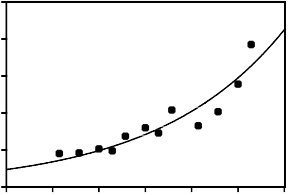


0 1 6 8 3 3 6 5 0 4 6 7 2 8 4 0 1 0 0 8

T im e [ h ]

4 0 0

**T u m o r V o lu m e ( m m 3 )**

3 0 0

2 0 0

1 0 0

0


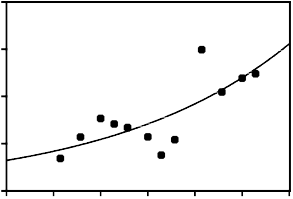


0 1 6 8 3 3 6 5 0 4 6 7 2 8 4 0 1 0 0 8

**T im e [ h ]**

1 5 0 0

T u m o r V o lu m e ( m m 3 )

1 0 0 0

5 0 0

0


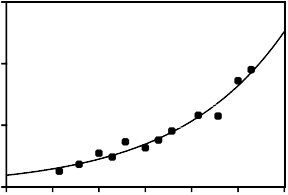


0 1 6 8 3 3 6 5 0 4 6 7 2 8 4 0 1 0 0 8

T im e [ h ]

1 5 0 0

**T u m o r V o lu m e ( m m 3 )**

1 0 0 0

5 0 0

0


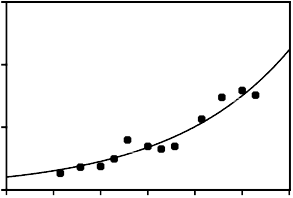


0 1 6 8 3 3 6 5 0 4 6 7 2 8 4 0 1 0 0 8

**T im e [ h ]**

1 0 0 0

T u m o r V o lu m e ( m m 3 )

8 0 0

6 0 0

4 0 0

2 0 0

0


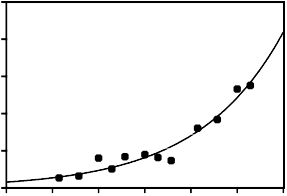


0 1 6 8 3 3 6 5 0 4 6 7 2 8 4 0 1 0 0 8

T im e [ h ]

3 0 0

**T u m o r V o lu m e ( m m 3 )**

2 0 0

1 0 0

0


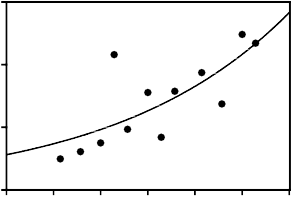


0 1 6 8 3 3 6 5 0 4 6 7 2 8 4 0 1 0 0 8

**T im e [ h ]**

**C U C R C 0 9 8 Ir in o te c a n**

**5 0 0**

**T u m o r V o lu m e ( m m 3 )**

**4 0 0**

**3 0 0**

**2 0 0**

**1 0 0**

**0**


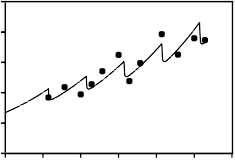


**0 1 6 8 3 3 6 5 0 4 6 7 2 8 4 0 1 0 0 8**

**T im e [ h ]**

**8 0 0**

**T u m o r V o lu m e ( m m 3 )**

**6 0 0**

**4 0 0**

**2 0 0**

**0**


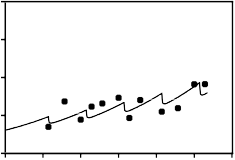


**0 1 6 8 3 3 6 5 0 4 6 7 2 8 4 0 1 0 0 8**

**T im e [ h ]**

**1 5 0 0**

**T u m o r V o lu m e ( m m 3 )**

**1 0 0 0**

**5 0 0**

**0**


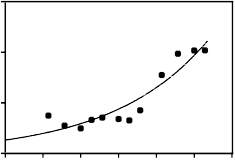


**0 1 6 8 3 3 6 5 0 4 6 7 2 8 4 0 1 0 0 8**

**T im e [ h ]**

**1 5 0 0**

**T u m o r V o lu m e ( m m 3 )**

**1 0 0 0**

**5 0 0**

**0**


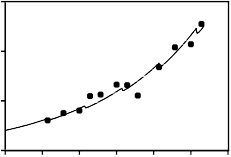


**0 1 6 8 3 3 6 5 0 4 6 7 2 8 4 0 1 0 0 8**

**T im e [ h ]**

**4 0 0**

**T u m o r V o lu m e ( m m 3 )**

**3 0 0**

**2 0 0**

**1 0 0**

**0**


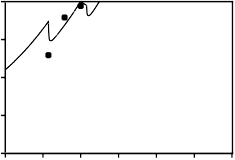


**0 1 6 8 3 3 6 5 0 4 6 7 2 8 4 0 1 0 0 8**

**T im e [ h ]**

**3 0 0**

**T u m o r V o lu m e ( m m 3 )**

**2 0 0**

**1 0 0**

**0**


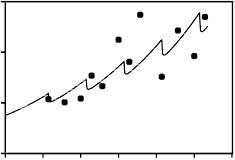


**0 1 6 8 3 3 6 5 0 4 6 7 2 8 4 0 1 0 0 8**

**T im e [ h ]**

**8 0 0**

**T u m o r V o lu m e ( m m 3 )**

**6 0 0**

**4 0 0**

**2 0 0**

**0**


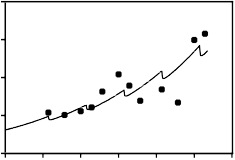


**0 1 6 8 3 3 6 5 0 4 6 7 2 8 4 0 1 0 0 8**

**T im e [ h ]**

**2 0 0 0**

**T u m o r V o lu m e ( m m 3 )**

**1 5 0 0**

**1 0 0 0**

**5 0 0**

**0**


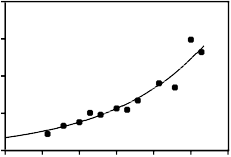


**0 1 6 8 3 3 6 5 0 4 6 7 2 8 4 0 1 0 0 8**

**T im e [ h ]**

**8 0 0**

**T u m o r V o lu m e ( m m 3 )**

**6 0 0**

**4 0 0**

**2 0 0**

**0**

**0 1 6 8 3 3 6 5 0 4 6 7 2 8 4 0 1 0 0 8**

**T im e [ h ]**

**5 0 0**

**T u m o r V o lu m e ( m m 3 )**

**4 0 0**

**3 0 0**

**2 0 0**

**1 0 0**

**0**

**0 1 6 8 3 3 6 5 0 4 6 7 2 8 4 0 1 0 0 8**

**T im e [ h ]**

**2 0 0 0**

**T u m o r V o lu m e ( m m 3 )**

**1 5 0 0**

**1 0 0 0**

**5 0 0**

**0**

**0 1 6 8 3 3 6 5 0 4 6 7 2 8 4 0 1 0 0 8**

**T im e [ h ]**

**2 5 0 0**

**T u m o r V o lu m e ( m m 3 )**

**2 0 0 0**

**1 5 0 0**

**1 0 0 0**

**5 0 0**

**0**

**0 1 6 8 3 3 6 5 0 4 6 7 2 8 4 0 1 0 0 8**

**T im e [ h ]**

**3 0 0**

**T u m o r V o lu m e ( m m 3 )**

**2 0 0**

**1 0 0**

**0**

**0 1 6 8 3 3 6 5 0 4 6 7 2 8 4 0 1 0 0 8**

**T im e [ h ]**

**6 0 0**

**T u m o r V o lu m e ( m m 3 )**

**4 0 0**

**2 0 0**

**0**

**0 1 6 8 3 3 6 5 0 4 6 7 2 8 4 0 1 0 0 8**

**T im e [ h ]**

**8 0 0**

**T u m o r V o lu m e ( m m 3 )**

**6 0 0**

**4 0 0**

**2 0 0**

**0**

**0 1 6 8 3 3 6 5 0 4 6 7 2 8 4 0 1 0 0 8**

**T im e [ h ]**

**6 0 0**

**T u m o r V o lu m e ( m m 3 )**

**4 0 0**

**2 0 0**

**0**

**0 1 6 8 3 3 6 5 0 4 6 7 2 8 4 0 1 0 0 8**

**T im e [ h ]**

**8 0 0**

**T u m o r V o lu m e ( m m 3 )**

**6 0 0**

**4 0 0**

**2 0 0**

**0**

**0 1 6 8 3 3 6 5 0 4 6 7 2 8 4 0 1 0 0 8**

**T im e [ h ]**

**2 0 0**

**T u m o r V o lu m e ( m m 3 )**

**1 5 0**

**1 0 0**

**5 0**

**0**

**0 1 6 8 3 3 6 5 0 4 6 7 2 8 4 0 1 0 0 8**

**T im e [ h ]**

## C U C R C 0 9 8 A l is e r t ib

4 0 0

**8 0 0**

**4 0 0**

**3 0 0**

**T u m o r V o lu m e ( m m 3 )**

**6 0 0**

**3 0 0**

**2 0 0**

**4 0 0**

**2 0 0**

**1 0 0**

**2 0 0**

**1 0 0**

**0**

**T u m o r V o lu m e ( m m 3 )**

**T u m o r V o lu m e ( m m 3 )**

**0 1 6 8 3 3 6 5 0 4 6 7 2 8 4 0 1 0 0 8**

**T im e [ h ]**

**0**

0 1 6 8 3 3 6 5 0 4 6 7 2 8 4 0 1 0 0 8

**T im e [ h ]**

**0**

0 1 6 8 3 3 6 5 0 4 6 7 2 8 4 0 1 0 0 8

**T im e [ h ]**

**1 0 0 0**

**1 5 0 0**

**1 5 0 0**

**8 0 0**

**T u m o r V o lu m e ( m m 3 )**

**T u m o r V o lu m e ( m m 3 )**

**T u m o r V o lu m e ( m m 3 )**

**6 0 0**

**1 0 0 0**

**1 0 0 0**

**4 0 0**

**5 0 0**

**5 0 0**

**2 0 0**

**0**

**0 1 6 8 3 3 6 5 0 4 6 7 2 8 4 0 1 0 0 8**

**T im e [ h ]**

**0**

0 1 6 8 3 3 6 5 0 4 6 7 2 8 4 0 1 0 0 8

**T im e [ h ]**

**0**

0 1 6 8 3 3 6 5 0 4 6 7 2 8 4 0 1 0 0 8

**T im e [ h ]**

**1 0 0 0**

**6 0 0**

**8 0 0**

**8 0 0**

**T u m o r V o lu m e ( m m 3 )**

**6 0 0**

**4 0 0**

**2 0 0**

**4 0 0**

**T u m o r V o lu m e ( m m 3 )**

**2 0 0**

**6 0 0**

**T u m o r V o lu m e ( m m 3 )**

**4 0 0**

**2 0 0**

**0**

**0 1 6 8 3 3 6 5 0 4 6 7 2 8 4 0 1 0 0 8**

**T im e [ h ]**

**0**

0 1 6 8 3 3 6 5 0 4 6 7 2 8 4 0 1 0 0 8

**T im e [ h ]**

**0**

0 1 6 8 3 3 6 5 0 4 6 7 2 8 4 0 1 0 0 8

**T im e [ h ]**

**C U C R C 0 9 8 C o m b in a t io n**

**8 0 0**

**T u m o r V o lu m e ( m m 3 )**

**6 0 0**

**4 0 0**

**2 0 0**

**0**

**0 1 6 8 3 3 6 5 0 4 6 7 2 8 4 0 1 0 0 8**

**T im e [ h ]**

**4 0 0**

**T u m o r V o lu m e ( m m 3 )**

**3 0 0**

**2 0 0**

**1 0 0**

**0**

**0 1 6 8 3 3 6 5 0 4 6 7 2 8 4 0 1 0 0 8**

**T im e [ h ]**

**4 0 0**

**T u m o r V o lu m e ( m m 3 )**

**3 0 0**

**2 0 0**

**1 0 0**

**0**

**0 1 6 8 3 3 6 5 0 4 6 7 2 8 4 0 1 0 0 8**

**T im e [ h ]**

**3 0 0**

**T u m o r V o lu m e ( m m 3 )**

**2 0 0**

**1 0 0**

**0**

**0 1 6 8 3 3 6 5 0 4 6 7 2 8 4 0 1 0 0 8**

**T im e [ h ]**

**5 0 0**

**T u m o r V o lu m e ( m m 3 )**

**4 0 0**

**3 0 0**

**2 0 0**

**1 0 0**

**0**

**0 1 6 8 3 3 6 5 0 4 6 7 2 8 4 0 1 0 0 8**

**T im e [ h ]**

**3 0 0**

**T u m o r V o lu m e ( m m 3 )**

**2 0 0**

**1 0 0**

**0**

**0 1 6 8 3 3 6 5 0 4 6 7 2 8 4 0 1 0 0 8**

**T im e [ h ]**

**8 0 0**

**T u m o r V o lu m e ( m m 3 )**

**6 0 0**

**4 0 0**

**2 0 0**

**0**

**0 1 6 8 3 3 6 5 0 4 6 7 2 8 4 0 1 0 0 8**

**T im e [ h ]**

**3 0 0**

**T u m o r V o lu m e ( m m 3 )**

**2 0 0**

**1 0 0**

**0**

**0 1 6 8 3 3 6 5 0 4 6 7 2 8 4 0 1 0 0 8**

**T im e [ h ]**

**8 0 0**

**T u m o r V o lu m e ( m m 3 )**

**6 0 0**

**4 0 0**

**2 0 0**

**0**

**0 1 6 8 3 3 6 5 0 4 6 7 2 8 4 0 1 0 0 8**

**T im e [ h ]**

**6 0 0**

**T u m o r V o lu m e ( m m 3 )**

**4 0 0**

**2 0 0**

**0**

**0 1 6 8 3 3 6 5 0 4 6 7 2 8 4 0 1 0 0 8**

**T im e [ h ]**

**5 0 0**

**T u m o r V o lu m e ( m m 3 )**

**4 0 0**

**3 0 0**

**2 0 0**

**1 0 0**

**0**

**0 1 6 8 3 3 6 5 0 4 6 7 2 8 4 0 1 0 0 8**

**T im e [ h ]**

**2 5 0**

**T u m o r V o lu m e ( m m 3 )**

**2 0 0**

**1 5 0**

**1 0 0**

**5 0**

**0**

**0 1 6 8 3 3 6 5 0 4 6 7 2 8 4 0 1 0 0 8**

**T im e [ h ]**

**5 0 0**

**T u m o r V o lu m e ( m m 3 )**

**4 0 0**

**3 0 0**

**2 0 0**

**1 0 0**

**0**

**0 1 6 8 3 3 6 5 0 4 6 7 2 8 4 0 1 0 0 8**

**T im e [ h ]**

**6 0 0**

**T u m o r V o lu m e ( m m 3 )**

**4 0 0**

**2 0 0**

**0**

**0 1 6 8 3 3 6 5 0 4 6 7 2 8 4 0 1 0 0 8**

**T im e [ h ]**

**1 5 0 0**

**T u m o r V o lu m e ( m m 3 )**

**1 0 0 0**

**5 0 0**

**0**

**0 1 6 8 3 3 6 5 0 4 6 7 2 8 4 0 1 0 0 8**

**T im e [ h ]**

**6 0 0**

**T u m o r V o lu m e ( m m 3 )**

**4 0 0**

**2 0 0**

**0**

**0 1 6 8 3 3 6 5 0 4 6 7 2 8 4 0 1 0 0 8**

**T im e [ h ]**

**8 0 0**

**T u m o r V o lu m e ( m m 3 )**

**6 0 0**

**4 0 0**

**2 0 0**

**0**

**0 1 6 8 3 3 6 5 0 4 6 7 2 8 4 0 1 0 0 8**

**T im e [ h ]**

**8 0 0**

**T u m o r V o lu m e ( m m 3 )**

**6 0 0**

**4 0 0**

**2 0 0**

**0**

**0 1 6 8 3 3 6 5 0 4 6 7 2 8 4 0 1 0 0 8**

**T im e [ h ]**

**1 0 0 0**

**T u m o r V o lu m e ( m m 3 )**

**8 0 0**

**6 0 0**

**4 0 0**

**2 0 0**

**0**

**0 1 6 8 3 3 6 5 0 4 6 7 2 8 4 0 1 0 0 8**

**T im e [ h ]**

# C U C R C 1 0 2 C o n t r o l

1 5 0 0

**T u m o r V o lu m e ( m m 3 )**

1 0 0 0

5 0 0

6 0 0

**T u m o r V o lu m e ( m m 3 )**

4 0 0

2 0 0

3 0 0 0

**T u m o r V o lu m e ( m m 3 )**

2 0 0 0

1 0 0 0

6 0 0

**T u m o r V o lu m e ( m m 3 )**

4 0 0

2 0 0

0

0 1 6 8 3 3 6 5 0 4 6 7 2 8 4 0 1 0 0 8 1 1 7 6 1 3 4 4

**T im e [ h ]**

0

0 1 6 8 3 3 6 5 0 4 6 7 2 8 4 0 1 0 0 8 1 1 7 6 1 3 4 4

**T im e [ h ]**

0

0 1 6 8 3 3 6 5 0 4 6 7 2 8 4 0 1 0 0 8 1 1 7 6 1 3 4 4

**T im e [ h ]**

0

0 1 6 8 3 3 6 5 0 4 6 7 2 8 4 0 1 0 0 8 1 1 7 6 1 3 4 4

**T im e [ h ]**

3 0 0 0

**T u m o r V o lu m e ( m m 3 )**

2 0 0 0

1 0 0 0

0

0 1 6 8 3 3 6 5 0 4 6 7 2 8 4 0 1 0 0 8 1 1 7 6 1 3 4 4

**T im e [ h ]**

6 0 0

**T u m o r V o lu m e ( m m 3 )**

4 0 0

2 0 0

0

0 1 6 8 3 3 6 5 0 4 6 7 2 8 4 0 1 0 0 8 1 1 7 6 1 3 4 4

**T im e [ h ]**

1 5 0 0

**T u m o r V o lu m e ( m m 3 )**

1 0 0 0

5 0 0

0

0 1 6 8 3 3 6 5 0 4 6 7 2 8 4 0 1 0 0 8 1 1 7 6 1 3 4 4

**T im e [ h ]**

1 5 0 0

**T u m o r V o lu m e ( m m 3 )**

1 0 0 0

5 0 0

0

0 1 6 8 3 3 6 5 0 4 6 7 2 8 4 0 1 0 0 8 1 1 7 6 1 3 4 4

**T im e [ h ]**

1 5 0 0

**T u m o r V o lu m e ( m m 3 )**

1 0 0 0

5 0 0

0

0 1 6 8 3 3 6 5 0 4 6 7 2 8 4 0 1 0 0 8 1 1 7 6 1 3 4 4

**T im e [ h ]**

1 5 0 0

**T u m o r V o lu m e ( m m 3 )**

1 0 0 0

5 0 0

0

0 1 6 8 3 3 6 5 0 4 6 7 2 8 4 0 1 0 0 8 1 1 7 6 1 3 4 4

**T im e [ h ]**

**C U C R C 1 0 2 Ir in o te c a n**

**8 0 0**

**T u m o r V o lu m e ( m m 3 )**

**6 0 0**

**4 0 0**

**2 0 0**

**0**

**0 1 6 8 3 3 6 5 0 4 6 7 2 8 4 0 1 0 0 8 1 1 7 6 1 3 4 4**

**T im e [ h ]**

**8 0 0**

**T u m o r V o lu m e ( m m 3 )**

**6 0 0**

**4 0 0**

**2 0 0**

**0**

**0 1 6 8 3 3 6 5 0 4 6 7 2 8 4 0 1 0 0 8 1 1 7 6 1 3 4 4**

**T im e [ h ]**

**4 0 0**

**T u m o r V o lu m e ( m m 3 )**

**3 0 0**

**2 0 0**

**1 0 0**

**0**

**0 1 6 8 3 3 6 5 0 4 6 7 2 8 4 0 1 0 0 8 1 1 7 6 1 3 4 4**

**T im e [ h ]**

**8 0 0**

**T u m o r V o lu m e ( m m 3 )**

**6 0 0**

**4 0 0**

**2 0 0**

**0**

**0 1 6 8 3 3 6 5 0 4 6 7 2 8 4 0 1 0 0 8 1 1 7 6 1 3 4 4**

**T im e [ h ]**

**4 0 0**

**T u m o r V o lu m e ( m m 3 )**

**3 0 0**

**2 0 0**

**1 0 0**

**0**

**0 1 6 8 3 3 6 5 0 4 6 7 2 8 4 0 1 0 0 8 1 1 7 6 1 3 4 4**

**T im e [ h ]**

**4 0 0**

**T u m o r V o lu m e ( m m 3 )**

**3 0 0**

**2 0 0**

**1 0 0**

**0**

**0 1 6 8 3 3 6 5 0 4 6 7 2 8 4 0 1 0 0 8 1 1 7 6 1 3 4 4**

**T im e [ h ]**

**2 0 0 0**

**T u m o r V o lu m e ( m m 3 )**

**1 5 0 0**

**1 0 0 0**

**5 0 0**

**0**

**0 1 6 8 3 3 6 5 0 4 6 7 2 8 4 0 1 0 0 8 1 1 7 6 1 3 4 4**

**T im e [ h ]**

**C U C R C 1 0 2 A l is e r t ib**

**4 0 0**

**T u m o r V o lu m e ( m m 3 )**

**3 0 0**

**2 0 0**

**1 0 0**

**0**

**0 1 6 8 3 3 6 5 0 4 6 7 2 8 4 0 1 0 0 8 1 1 7 6 1 3 4 4**

**T im e [ h ]**

**1 5 0 0**

**T u m o r V o lu m e ( m m 3 )**

**1 0 0 0**

**5 0 0**

**0**

**0 1 6 8 3 3 6 5 0 4 6 7 2 8 4 0 1 0 0 8 1 1 7 6 1 3 4 4**

**T im e [ h ]**

**4 0 0**

**T u m o r V o lu m e ( m m 3 )**

**3 0 0**

**2 0 0**

**1 0 0**

**0**

**0 1 6 8 3 3 6 5 0 4 6 7 2 8 4 0 1 0 0 8 1 1 7 6 1 3 4 4**

**T im e [ h ]**

**1 5 0 0**

**T u m o r V o lu m e ( m m 3 )**

**1 0 0 0**

**5 0 0**

**0**

**0 1 6 8 3 3 6 5 0 4 6 7 2 8 4 0 1 0 0 8 1 1 7 6 1 3 4 4**

**T im e [ h ]**

**4 0 0**

**T u m o r V o lu m e ( m m 3 )**

**2 0 0**

**0**

**0 1 6 8 3 3 6 5 0 4 6 7 2 8 4 0 1 0 0 8 1 1 7 6 1 3 4 4**

**T im e [ h ]**

**4 0 0**

**T u m o r V o lu m e ( m m 3 )**

**3 0 0**

**2 0 0**

**1 0 0**

**0**

**0 1 6 8 3 3 6 5 0 4 6 7 2 8 4 0 1 0 0 8 1 1 7 6 1 3 4 4**

**T im e [ h ]**

**1 5 0 0**

**T u m o r V o lu m e ( m m 3 )**

**1 0 0 0**

**5 0 0**

**0**

**0 1 6 8 3 3 6 5 0 4 6 7 2 8 4 0 1 0 0 8 1 1 7 6 1 3 4 4**

**T im e [ h ]**

**4 0 0**

**T u m o r V o lu m e ( m m 3 )**

**3 0 0**

**2 0 0**

**1 0 0**

**0**

**0 1 6 8 3 3 6 5 0 4 6 7 2 8 4 0 1 0 0 8 1 1 7 6 1 3 4 4**

**T im e [ h ]**

**3 0 0 0**

**T u m o r V o lu m e ( m m 3 )**

**2 0 0 0**

**1 0 0 0**

**0**

**0 1 6 8 3 3 6 5 0 4 6 7 2 8 4 0 1 0 0 8 1 1 7 6 1 3 4 4**

**T im e [ h ]**

**C U C R C 1 0 2 C o m b in a t io n**

**1 5 0 0**

**T u m o r V o lu m e ( m m 3 )**

**1 0 0 0**

**5 0 0**

**0**

**0 1 6 8 3 3 6 5 0 4 6 7 2 8 4 0 1 0 0 8 1 1 7 6 1 3 4 4**

**T im e [ h ]**

**1 5 0 0**

**T u m o r V o lu m e ( m m 3 )**

**1 0 0 0**

**5 0 0**

**0**

**0 1 6 8 3 3 6 5 0 4 6 7 2 8 4 0 1 0 0 8 1 1 7 6 1 3 4 4**

**T im e [ h ]**

**3 0 0**

**T u m o r V o lu m e ( m m 3 )**

**2 0 0**

**1 0 0**

**0**

**0 1 6 8 3 3 6 5 0 4 6 7 2 8 4 0 1 0 0 8 1 1 7 6 1 3 4 4**

**T im e [ h ]**

**3 0 0**

**T u m o r V o lu m e ( m m 3 )**

**2 0 0**

**1 0 0**

**0**

**0 1 6 8 3 3 6 5 0 4 6 7 2 8 4 0 1 0 0 8 1 1 7 6 1 3 4 4**

**T im e [ h ]**

**3 0 0**

**T u m o r V o lu m e ( m m 3 )**

**2 0 0**

**1 0 0**

**0**

**0 1 6 8 3 3 6 5 0 4 6 7 2 8 4 0 1 0 0 8 1 1 7 6 1 3 4 4**

**T im e [ h ]**

**3 0 0**

**T u m o r V o lu m e ( m m 3 )**

**2 0 0**

**1 0 0**

**0**

**0 1 6 8 3 3 6 5 0 4 6 7 2 8 4 0 1 0 0 8 1 1 7 6 1 3 4 4**

**T im e [ h ]**

**3 0 0**

**T u m o r V o lu m e ( m m 3 )**

**2 0 0**

**1 0 0**

**0**

**0 1 6 8 3 3 6 5 0 4 6 7 2 8 4 0 1 0 0 8 1 1 7 6 1 3 4 4**

**T im e [ h ]**

**6 0 0**

**T u m o r V o lu m e ( m m 3 )**

**4 0 0**

**2 0 0**

**0**

**0 1 6 8 3 3 6 5 0 4 6 7 2 8 4 0 1 0 0 8 1 1 7 6 1 3 4 4**

**T im e [ h ]**

**C U C R C 1 0 8 C o n t r o l**

8 0 0

T u m o r V o lu m e ( m m 3 )

6 0 0

4 0 0

2 0 0

0

0 1 6 8 3 3 6 5 0 4 6 7 2 8 4 0 1 0 0 8

T im e [ h ]

**3 0 0 0**

T u m o r V o lu m e ( m m 3 )

**2 0 0 0**

**1 0 0 0**

**0**

**0 1 6 8 3 3 6 5 0 4 6 7 2 8 4 0 1 0 0 8**

T im e [ h ]

**1 5 0 0**

T u m o r V o lu m e ( m m 3 )

**1 0 0 0**

**5 0 0**

**0**

**0 1 6 8 3 3 6 5 0 4 6 7 2 8 4 0 1 0 0 8**

T im e [ h ]

4 0 0

T u m o r V o lu m e ( m m 3 )

3 0 0

2 0 0

1 0 0

0

0 1 6 8 3 3 6 5 0 4 6 7 2 8 4 0 1 0 0 8

T im e [ h ]

**1 0 0 0**

T u m o r V o lu m e ( m m 3 )

**8 0 0**

**6 0 0**

**4 0 0**

**2 0 0**

**0**

**0 1 6 8 3 3 6 5 0 4 6 7 2 8 4 0 1 0 0 8**

T im e [ h ]

**1 5 0 0**

T u m o r V o lu m e ( m m 3 )

**1 0 0 0**

**5 0 0**

**0**

**0 1 6 8 3 3 6 5 0 4 6 7 2 8 4 0 1 0 0 8**

T im e [ h ]

3 0 0

T u m o r V o lu m e ( m m 3 )

2 0 0

1 0 0

0

0 1 6 8 3 3 6 5 0 4 6 7 2 8 4 0 1 0 0 8

T im e [ h ]

**2 0 0 0**

T u m o r V o lu m e ( m m 3 )

**1 5 0 0**

**1 0 0 0**

**5 0 0**

**0**

**0 1 6 8 3 3 6 5 0 4 6 7 2 8 4 0 1 0 0 8**

T im e [ h ]

8 0 0

T u m o r V o lu m e ( m m 3 )

6 0 0

4 0 0

2 0 0

0

0 1 6 8 3 3 6 5 0 4 6 7 2 8 4 0 1 0 0 8

T im e [ h ]

8 0 0

T u m o r V o lu m e ( m m 3 )

6 0 0

4 0 0

2 0 0

0

0 1 6 8 3 3 6 5 0 4 6 7 2 8 4 0 1 0 0 8

T im e [ h ]

**2 0 0 0**

T u m o r V o lu m e ( m m 3 )

**1 5 0 0**

**1 0 0 0**

**5 0 0**

**0**

**0 1 6 8 3 3 6 5 0 4 6 7 2 8 4 0 1 0 0 8**

T im e [ h ]

3 0 0

T u m o r V o lu m e ( m m 3 )

2 0 0

1 0 0

0

0 1 6 8 3 3 6 5 0 4 6 7 2 8 4 0 1 0 0 8

T im e [ h ]

**4 0 0**

T u m o r V o lu m e ( m m 3 )

**3 0 0**

**2 0 0**

**1 0 0**

**0**

**0 1 6 8 3 3 6 5 0 4 6 7 2 8 4 0 1 0 0 8**

T im e [ h ]

5 0 0

T u m o r V o lu m e ( m m 3 )

4 0 0

3 0 0

2 0 0

1 0 0

0

0 1 6 8 3 3 6 5 0 4 6 7 2 8 4 0 1 0 0 8

T im e [ h ]

**2 0 0 0**

T u m o r V o lu m e ( m m 3 )

**1 5 0 0**

**1 0 0 0**

**5 0 0**

**0**

**0 1 6 8 3 3 6 5 0 4 6 7 2 8 4 0 1 0 0 8**

T im e [ h ]

2 0 0

T u m o r V o lu m e ( m m 3 )

1 5 0

1 0 0

5 0

0

0 1 6 8 3 3 6 5 0 4 6 7 2 8 4 0 1 0 0 8

T im e [ h ]

**C U C R C 1 0 8 Ir in o te c a n**

4 0 0

**T u m o r V o lu m e ( m m 3 )**

3 0 0

2 0 0

1 0 0

0

0 1 6 8 3 3 6 5 0 4 6 7 2 8 4 0 1 0 0 8

**T im e [ h ]**

8 0 0

**T u m o r V o lu m e ( m m 3 )**

6 0 0

4 0 0

2 0 0

0

0 1 6 8 3 3 6 5 0 4 6 7 2 8 4 0 1 0 0 8

**T im e [ h ]**

4 0 0

**T u m o r V o lu m e ( m m 3 )**

3 0 0

2 0 0

1 0 0

0

0 1 6 8 3 3 6 5 0 4 6 7 2 8 4 0 1 0 0 8

**T im e [ h ]**

1 5 0 0

**T u m o r V o lu m e ( m m 3 )**

1 0 0 0

5 0 0

0

0 1 6 8 3 3 6 5 0 4 6 7 2 8 4 0 1 0 0 8

**T im e [ h ]**

4 0 0

**T u m o r V o lu m e ( m m 3 )**

3 0 0

2 0 0

1 0 0

0

0 1 6 8 3 3 6 5 0 4 6 7 2 8 4 0 1 0 0 8

**T im e [ h ]**

8 0 0

**T u m o r V o lu m e ( m m 3 )**

6 0 0

4 0 0

2 0 0

0

0 1 6 8 3 3 6 5 0 4 6 7 2 8 4 0 1 0 0 8

**T im e [ h ]**

8 0

**T u m o r V o lu m e ( m m 3 )**

6 0

4 0

2 0

0

0 1 6 8 3 3 6 5 0 4 6 7 2 8 4 0 1 0 0 8

**T im e [ h ]**

2 5 0

**T u m o r V o lu m e ( m m 3 )**

2 0 0

1 5 0

1 0 0

5 0

0

0 1 6 8 3 3 6 5 0 4 6 7 2 8 4 0 1 0 0 8

**T im e [ h ]**

4 0 0

**T u m o r V o lu m e ( m m 3 )**

3 0 0

2 0 0

1 0 0

0

0 1 6 8 3 3 6 5 0 4 6 7 2 8 4 0 1 0 0 8

**T im e [ h ]**

2 5 0

**T u m o r V o lu m e ( m m 3 )**

2 0 0

1 5 0

1 0 0

5 0

0

0 1 6 8 3 3 6 5 0 4 6 7 2 8 4 0 1 0 0 8

**T im e [ h ]**

8 0 0

**T u m o r V o lu m e ( m m 3 )**

6 0 0

4 0 0

2 0 0

0

0 1 6 8 3 3 6 5 0 4 6 7 2 8 4 0 1 0 0 8

**T im e [ h ]**

2 0 0 0

**T u m o r V o lu m e ( m m 3 )**

1 5 0 0

1 0 0 0

5 0 0

0

0 1 6 8 3 3 6 5 0 4 6 7 2 8 4 0 1 0 0 8

**T im e [ h ]**

8 0 0

**T u m o r V o lu m e ( m m 3 )**

6 0 0

4 0 0

2 0 0

0

0 1 6 8 3 3 6 5 0 4 6 7 2 8 4 0 1 0 0 8

**T im e [ h ]**

4 0 0

**T u m o r V o lu m e ( m m 3 )**

3 0 0

2 0 0

1 0 0

0

0 1 6 8 3 3 6 5 0 4 6 7 2 8 4 0 1 0 0 8

**T im e [ h ]**

2 0 0

**T u m o r V o lu m e ( m m 3 )**

1 5 0

1 0 0

5 0

0

0 1 6 8 3 3 6 5 0 4 6 7 2 8 4 0 1 0 0 8

**T im e [ h ]**

1 5 0 0

**T u m o r V o lu m e ( m m 3 )**

1 0 0 0

5 0 0

0

0 1 6 8 3 3 6 5 0 4 6 7 2 8 4 0 1 0 0 8

**T im e [ h ]**

**C U C R C 1 0 8 A l is e r t ib**

4 0 0

**T u m o r V o lu m e ( m m 3 )**

3 0 0

2 0 0

1 0 0

0

0 1 6 8 3 3 6 5 0 4 6 7 2 8 4 0 1 0 0 8

**T im e [ h ]**

4 0 0

**T u m o r V o lu m e ( m m 3 )**

3 0 0

2 0 0

1 0 0

0

0 1 6 8 3 3 6 5 0 4 6 7 2 8 4 0 1 0 0 8

**T im e [ h ]**

4 0 0

**T u m o r V o lu m e ( m m 3 )**

3 0 0

2 0 0

1 0 0

0

0 1 6 8 3 3 6 5 0 4 6 7 2 8 4 0 1 0 0 8

**T im e [ h ]**

6 0 0

**T u m o r V o lu m e ( m m 3 )**

4 0 0

2 0 0

0

0 1 6 8 3 3 6 5 0 4 6 7 2 8 4 0 1 0 0 8

**T im e [ h ]**

4 0 0

**T u m o r V o lu m e ( m m 3 )**

2 0 0

0

0 1 6 8 3 3 6 5 0 4 6 7 2 8 4 0 1 0 0 8

**T im e [ h ]**

2 5 0

**T u m o r V o lu m e ( m m 3 )**

2 0 0

1 5 0

1 0 0

5 0

0

0 1 6 8 3 3 6 5 0 4 6 7 2 8 4 0 1 0 0 8

**T im e [ h ]**

5 0 0

**T u m o r V o lu m e ( m m 3 )**

4 0 0

3 0 0

2 0 0

1 0 0

0

0 1 6 8 3 3 6 5 0 4 6 7 2 8 4 0 1 0 0 8

**T im e [ h ]**

1 5 0

**T u m o r V o lu m e ( m m 3 )**

1 0 0

5 0

0

0 1 6 8 3 3 6 5 0 4 6 7 2 8 4 0 1 0 0 8

**T im e [ h ]**

2 0 0

**T u m o r V o lu m e ( m m 3 )**

1 5 0

1 0 0

5 0

0

0 1 6 8 3 3 6 5 0 4 6 7 2 8 4 0 1 0 0 8

**T im e [ h ]**

4 0

**T u m o r V o lu m e ( m m 3 )**

3 0

2 0

1 0

0

0 1 6 8 3 3 6 5 0 4 6 7 2 8 4 0 1 0 0 8

**T im e [ h ]**

1 0 0

**T u m o r V o lu m e ( m m 3 )**

8 0

6 0

4 0

2 0

0

0 1 6 8 3 3 6 5 0 4 6 7 2 8 4 0 1 0 0 8

**T im e [ h ]**

3 0 0

**T u m o r V o lu m e ( m m 3 )**

2 0 0

1 0 0

0

0 1 6 8 3 3 6 5 0 4 6 7 2 8 4 0 1 0 0 8

**T im e [ h ]**

3 0 0

**T u m o r V o lu m e ( m m 3 )**

2 5 0

2 0 0

1 5 0

1 0 0

0 1 6 8 3 3 6 5 0 4 6 7 2 8 4 0 1 0 0 8

**T im e [ h ]**

6 0 0

**T u m o r V o lu m e ( m m 3 )**

4 0 0

2 0 0

0

0 1 6 8 3 3 6 5 0 4 6 7 2 8 4 0 1 0 0 8

**T im e [ h ]**

2 5 0

**T u m o r V o lu m e ( m m 3 )**

2 0 0

1 5 0

1 0 0

5 0

0

0 1 6 8 3 3 6 5 0 4 6 7 2 8 4 0 1 0 0 8

**T im e [ h ]**

2 5 0

**T u m o r V o lu m e ( m m 3 )**

2 0 0

1 5 0

1 0 0

5 0

0

0 1 6 8 3 3 6 5 0 4 6 7 2 8 4 0 1 0 0 8

**T im e [ h ]**

**C U C R C 1 0 8 C o m b in a t io n**

5 0

**T u m o r V o lu m e ( m m 3 )**

4 0

3 0

2 0

1 0

0

0 1 6 8 3 3 6 5 0 4 6 7 2 8 4 0 1 0 0 8

**T im e [ h ]**

8 0 0

**T u m o r V o lu m e ( m m 3 )**

6 0 0

4 0 0

2 0 0

0

0 1 6 8 3 3 6 5 0 4 6 7 2 8 4 0 1 0 0 8

**T im e [ h ]**

1 5 0

**T u m o r V o lu m e ( m m 3 )**

1 0 0

5 0

0

0 1 6 8 3 3 6 5 0 4 6 7 2 8 4 0 1 0 0 8

**T im e [ h ]**

2 5 0

**T u m o r V o lu m e ( m m 3 )**

2 0 0

1 5 0

1 0 0

5 0

0

0 1 6 8 3 3 6 5 0 4 6 7 2 8 4 0 1 0 0 8

**T im e [ h ]**

3 0 0

**T u m o r V o lu m e ( m m 3 )**

2 0 0

1 0 0

0

0 1 6 8 3 3 6 5 0 4 6 7 2 8 4 0 1 0 0 8

**T im e [ h ]**

1 0 0 0

**T u m o r V o lu m e ( m m 3 )**

8 0 0

6 0 0

4 0 0

2 0 0

0

0 1 6 8 3 3 6 5 0 4 6 7 2 8 4 0 1 0 0 8

**T im e [ h ]**

4 0 0

**T u m o r V o lu m e ( m m 3 )**

3 0 0

2 0 0

1 0 0

0

0 1 6 8 3 3 6 5 0 4 6 7 2 8 4 0 1 0 0 8

**T im e [ h ]**

6 0

**T u m o r V o lu m e ( m m 3 )**

4 0

2 0

0

0 1 6 8 3 3 6 5 0 4 6 7 2 8 4 0 1 0 0 8

**T im e [ h ]**

1 0 0 0

**T u m o r V o lu m e ( m m 3 )**

8 0 0

6 0 0

4 0 0

2 0 0

0

0 1 6 8 3 3 6 5 0 4 6 7 2 8 4 0 1 0 0 8

**T im e [ h ]**

2 0 0

**T u m o r V o lu m e ( m m 3 )**

1 5 0

1 0 0

5 0

0

0 1 6 8 3 3 6 5 0 4 6 7 2 8 4 0 1 0 0 8

**T im e [ h ]**

4 0 0

**T u m o r V o lu m e ( m m 3 )**

3 0 0

2 0 0

1 0 0

0

0 1 6 8 3 3 6 5 0 4 6 7 2 8 4 0 1 0 0 8

**T im e [ h ]**

1 5 0

**T u m o r V o lu m e ( m m 3 )**

1 0 0

5 0

0

0 1 6 8 3 3 6 5 0 4 6 7 2 8 4 0 1 0 0 8

**T im e [ h ]**

1 0 0

**T u m o r V o lu m e ( m m 3 )**

8 0

6 0

4 0

2 0

0

0 1 6 8 3 3 6 5 0 4 6 7 2 8 4 0 1 0 0 8

**T im e [ h ]**

**C U C R C 0 1 0 C o n t r o l**

**1 0 0 0**

**1 0 0 0**

**1 0 0 0**

**8 0 0**

**T u m o r V o lu m e ( m m 3 )**

**8 0 0**

**T u m o r V o lu m e ( m m 3 )**

**8 0 0**

**T u m o r V o lu m e ( m m 3 )**

**6 0 0**

**6 0 0**

**6 0 0**

**4 0 0**

**4 0 0**

**4 0 0**

**2 0 0**

**2 0 0**

**2 0 0**

**0**

**0 1 6 8 3 3 6 5 0 4 6 7 2 8 4 0**

**T im e [ h ]**

**0**

**0 1 6 8 3 3 6 5 0 4 6 7 2 8 4 0**

**T im e [ h ]**

**0**

**0 1 6 8 3 3 6 5 0 4 6 7 2 8 4 0**

**T im e [ h ]**

**1 0 0 0**

**4 0 0**

**6 0 0**

**8 0 0**

**T u m o r V o lu m e ( m m 3 )**

**6 0 0**

**4 0 0**

**2 0 0**

**3 0 0**

**T u m o r V o lu m e ( m m 3 )**

**2 0 0**

**1 0 0**

**4 0 0**

**T u m o r V o lu m e ( m m 3 )**

**2 0 0**

**0**

**0 1 6 8 3 3 6 5 0 4 6 7 2 8 4 0**

**T im e [ h ]**

**0**

**0 1 6 8 3 3 6 5 0 4 6 7 2 8 4 0**

**T im e [ h ]**

**0**

0 1 6 8 3 3 6 5 0 4 6 7 2 8 4 0

**T im e [ h ]**

**4 0 0**

**2 5 0 0**

**1 5 0 0**

**3 0 0**

**T u m o r V o lu m e ( m m 3 )**

**2 0 0**

**1 0 0**

**2 0 0 0**

**1 5 0 0**

**T u m o r V o lu m e ( m m 3 )**

**1 0 0 0**

**5 0 0**

**1 0 0 0**

**T u m o r V o lu m e ( m m 3 )**

**5 0 0**

**0**

**0 1 6 8 3 3 6 5 0 4 6 7 2 8 4 0**

**0**

0 1 6 8 3 3 6 5 0 4 6 7 2 8 4 0

**0**

0 1 6 8 3 3 6 5 0 4 6 7 2 8 4 0

**C U C R C 0 1 0 C e tu x im a b**

**4 0 0**

**1 0 0 0**

**4 0 0**

**3 0 0**

**T u m o r V o lu m e ( m m 3 )**

**8 0 0**

**3 0 0**

**T u m o r V o lu m e ( m m 3 )**

**2 0 0**

**6 0 0**

**4 0 0**

**2 0 0**

**1 0 0**

**2 0 0**

**1 0 0**

**0**

**T u m o r V o lu m e ( m m 3 )**

**0 1 6 8 3 3 6 5 0 4 6 7 2 8 4 0**

**T im e [ h ]**

**0**

0 1 6 8 3 3 6 5 0 4 6 7 2 8 4 0

**T im e [ h ]**

**0**

0 1 6 8 3 3 6 5 0 4 6 7 2 8 4 0

**T im e [ h ]**

**1 5 0 0**

**4 0 0**

**4 0 0**

**1 0 0 0**

**T u m o r V o lu m e ( m m 3 )**

**3 0 0**

**3 0 0**

**2 0 0**

**2 0 0**

**5 0 0**

**1 0 0**

**1 0 0**

**0**

**T u m o r V o lu m e ( m m 3 )**

**T u m o r V o lu m e ( m m 3 )**

**0 1 6 8 3 3 6 5 0 4 6 7 2 8 4 0**

**T im e [ h ]**

**0**

0 1 6 8 3 3 6 5 0 4 6 7 2 8 4 0

**T im e [ h ]**

**0**

0 1 6 8 3 3 6 5 0 4 6 7 2 8 4 0

**T im e [ h ]**

**4 0 0**

**4 0 0**

**4 0 0**

**3 0 0**

**T u m o r V o lu m e ( m m 3 )**

**3 0 0**

**3 0 0**

**2 0 0**

**2 0 0**

**2 0 0**

**1 0 0**

**1 0 0**

**1 0 0**

**0**

**T u m o r V o lu m e ( m m 3 )**

**T u m o r V o lu m e ( m m 3 )**

**0 1 6 8 3 3 6 5 0 4 6 7 2 8 4 0**

**0**

0 1 6 8 3 3 6 5 0 4 6 7 2 8 4 0

**0**

0 1 6 8 3 3 6 5 0 4 6 7 2 8 4 0

**C U C R C 0 1 0 A l is e r t ib**

1 0 0 0

**T u m o r V o lu m e ( m m 3 )**

8 0 0

6 0 0

4 0 0

2 0 0

0

0 1 6 8 3 3 6 5 0 4 6 7 2 8 4 0

**T im e [ h ]**

4 0 0

**T u m o r V o lu m e ( m m 3 )**

3 0 0

2 0 0

1 0 0

0

0 1 6 8 3 3 6 5 0 4 6 7 2 8 4 0

**T im e [ h ]**

1 0 0 0

**T u m o r V o lu m e ( m m 3 )**

8 0 0

6 0 0

4 0 0

2 0 0

0 1 6 8 3 3 6 5 0 4 6 7 2 8 4 0

**T im e [ h ]**

1 0 0 0

**T u m o r V o lu m e ( m m 3 )**

8 0 0

6 0 0

4 0 0

2 0 0

0 1 6 8 3 3 6 5 0 4 6 7 2 8 4 0

**T im e [ h ]**

4 0 0

**T u m o r V o lu m e ( m m 3 )**

3 0 0

2 0 0

1 0 0

1 0 0 0

**T u m o r V o lu m e ( m m 3 )**

8 0 0

6 0 0

4 0 0

2 0 0

2 0 0 0

**T u m o r V o lu m e ( m m 3 )**

1 5 0 0

1 0 0 0

5 0 0

4 0 0

**0**

**1 6 8 3 3 6 5 0 4 6 7 2 8 4 0**

**T u m o r V o lu m e ( m m 3 )**

3 0 0

2 0 0

1 0 0

0

0 1 6 8 3 3 6 5 0 4 6 7 2 8 4 0

**T im e [ h ]**

0

0 1 6 8 3 3 6 5 0 4 6 7 2 8 4 0

**T im e [ h ]**

0

0 1 6 8 3 3 6 5 0 4 6 7 2 8 4 0

**T im e [ h ]**

0

**T im e [ h ]**

4 0 0

**T u m o r V o lu m e ( m m 3 )**

3 0 0

2 0 0

1 0 0

0 1 6 8 3 3 6 5 0 4 6 7 2 8 4 0

**T im e [ h ]**

1 5 0 0

**T u m o r V o lu m e ( m m 3 )**

1 0 0 0

5 0 0

0

0 1 6 8 3 3 6 5 0 4 6 7 2 8 4 0

**T im e [ h ]**

**C U C R C 0 1 0 C o m b in a t io n**

4 0 0

**T u m o r V o lu m e ( m m 3 )**

3 0 0

2 0 0

1 0 0

0

0 1 6 8 3 3 6 5 0 4 6 7 2 8 4 0

**T im e [ h ]**

4 0 0

**T u m o r V o lu m e ( m m 3 )**

3 0 0

2 0 0

1 0 0

0

0 1 6 8 3 3 6 5 0 4 6 7 2 8 4 0

**T im e [ h ]**

4 0 0

**T u m o r V o lu m e ( m m 3 )**

3 0 0

2 0 0

1 0 0

0

0 1 6 8 3 3 6 5 0 4 6 7 2 8 4 0

**T im e [ h ]**

4 0 0

**T u m o r V o lu m e ( m m 3 )**

3 0 0

2 0 0

1 0 0

0

0 1 6 8 3 3 6 5 0 4 6 7 2 8 4 0

**T im e [ h ]**

4 0 0

**T u m o r V o lu m e ( m m 3 )**

3 0 0

2 0 0

1 0 0

0

0 1 6 8 3 3 6 5 0 4 6 7 2 8 4 0

**T im e [ h ]**

1 0 0 0

**T u m o r V o lu m e ( m m 3 )**

8 0 0

6 0 0

4 0 0

2 0 0

0

0 1 6 8 3 3 6 5 0 4 6 7 2 8 4 0

**T im e [ h ]**

4 0 0

**T u m o r V o lu m e ( m m 3 )**

3 0 0

2 0 0

1 0 0

0

0 1 6 8 3 3 6 5 0 4 6 7 2 8 4 0

**T im e [ h ]**

1 0 0 0

**T u m o r V o lu m e ( m m 3 )**

8 0 0

6 0 0

4 0 0

2 0 0

0

0 1 6 8 3 3 6 5 0 4 6 7 2 8 4 0

**T im e [ h ]**

4 0 0

**T u m o r V o lu m e ( m m 3 )**

3 0 0

2 0 0

1 0 0

0

0 1 6 8 3 3 6 5 0 4 6 7 2 8 4 0

**T im e [ h ]**

4 0 0

**T u m o r V o lu m e ( m m 3 )**

3 0 0

2 0 0

1 0 0

0

0 1 6 8 3 3 6 5 0 4 6 7 2 8 4 0

**T im e [ h ]**

**C U C R C 0 2 6 C o n t r o l**

**1 0 0 0**

**1 0 0 0**

**1 0 0 0**

**8 0 0**

**T u m o r V o lu m e ( m m 3 )**

**8 0 0**

**T u m o r V o lu m e ( m m 3 )**

**8 0 0**

**T u m o r V o lu m e ( m m 3 )**

**6 0 0**

**6 0 0**

**6 0 0**

**4 0 0**

**4 0 0**

**4 0 0**

**2 0 0**

**2 0 0**

**2 0 0**

**0**

**0 1 6 8 3 3 6 5 0 4 6 7 2 8 4 0**

**T im e [ h ]**

**0**

**0 1 6 8 3 3 6 5 0 4 6 7 2 8 4 0**

**T im e [ h ]**

**0**

**0 1 6 8 3 3 6 5 0 4 6 7 2 8 4 0**

**T im e [ h ]**

**1 0 0 0**

**4 0 0**

**6 0 0**

**8 0 0**

**T u m o r V o lu m e ( m m 3 )**

**6 0 0**

**4 0 0**

**2 0 0**

**3 0 0**

**T u m o r V o lu m e ( m m 3 )**

**2 0 0**

**1 0 0**

**4 0 0**

**T u m o r V o lu m e ( m m 3 )**

**2 0 0**

**0**

**0 1 6 8 3 3 6 5 0 4 6 7 2 8 4 0**

**T im e [ h ]**

**0**

0 1 6 8 3 3 6 5 0 4 6 7 2 8 4 0

**T im e [ h ]**

**0**

0 1 6 8 3 3 6 5 0 4 6 7 2 8 4 0

**T im e [ h ]**

**4 0 0**

**2 5 0 0**

**1 5 0 0**

**3 0 0**

**T u m o r V o lu m e ( m m 3 )**

**2 0 0**

**1 0 0**

**2 0 0 0**

**1 5 0 0**

**T u m o r V o lu m e ( m m 3 )**

**1 0 0 0**

**5 0 0**

**1 0 0 0**

**T u m o r V o lu m e ( m m 3 )**

**5 0 0**

**0**

**0 1 6 8 3 3 6 5 0 4 6 7 2 8 4 0**

**T im e [ h ]**

**0**

0 1 6 8 3 3 6 5 0 4 6 7 2 8 4 0

**T im e [ h ]**

**0**

0 1 6 8 3 3 6 5 0 4 6 7 2 8 4 0

**T im e [ h ]**

**C U C R C 0 2 6 C e tu x im a b**

2 5 0 0

**T u m o r V o lu m e ( m m 3 )**

2 0 0 0

1 5 0 0

1 0 0 0

5 0 0

0

0 1 6 8 3 3 6 5 0 4 6 7 2

**T im e [ h ]**

8 0 0

**T u m o r V o lu m e ( m m 3 )**

6 0 0

4 0 0

2 0 0

0

0 1 6 8 3 3 6 5 0 4 6 7 2

**T im e [ h ]**

8 0 0

**T u m o r V o lu m e ( m m 3 )**

6 0 0

4 0 0

2 0 0

0

0 1 6 8 3 3 6 5 0 4 6 7 2

**T im e [ h ]**

1 0 0 0

**T u m o r V o lu m e ( m m 3 )**

8 0 0

6 0 0

4 0 0

2 0 0

0

0 1 6 8 3 3 6 5 0 4 6 7 2

**T im e [ h ]**

2 5 0 0

**T u m o r V o lu m e ( m m 3 )**

2 0 0 0

1 5 0 0

1 0 0 0

5 0 0

0

0 1 6 8 3 3 6 5 0 4 6 7 2

**T im e [ h ]**

8 0 0

**T u m o r V o lu m e ( m m 3 )**

6 0 0

4 0 0

2 0 0

0

0 1 6 8 3 3 6 5 0 4 6 7 2

**T im e [ h ]**

1 0 0 0

**T u m o r V o lu m e ( m m 3 )**

8 0 0

6 0 0

4 0 0

2 0 0

0

0 1 6 8 3 3 6 5 0 4 6 7 2

**T im e [ h ]**

2 5 0 0

**T u m o r V o lu m e ( m m 3 )**

2 0 0 0

1 5 0 0

1 0 0 0

5 0 0

0

0 1 6 8 3 3 6 5 0 4 6 7 2

**T im e [ h ]**

8 0 0

**T u m o r V o lu m e ( m m 3 )**

6 0 0

4 0 0

2 0 0

0

0 1 6 8 3 3 6 5 0 4 6 7 2

**T im e [ h ]**

**8 0 0**

**T u m o r V o lu m e ( m m 3 )**

**6 0 0**

**4 0 0**

**2 0 0**

**0**

**0 1 6 8 3 3 6 5 0 4 6 7 2**

**T im e [ h ]**

**C U C R C 0 2 6 A l is e r t ib**

4 0 0

**T u m o r V o lu m e ( m m 3 )**

3 0 0

2 0 0

1 0 0

0

0 2 0 0 4 0 0 6 0 0

**T im e [ h ]**

4 0 0

**T u m o r V o lu m e ( m m 3 )**

3 0 0

2 0 0

1 0 0

0

0 1 6 8 3 3 6 5 0 4 6 7 2

**T im e [ h ]**

4 0 0

**T u m o r V o lu m e ( m m 3 )**

3 0 0

2 0 0

1 0 0

0

0 1 6 8 3 3 6 5 0 4 6 7 2

**T im e [ h ]**

4 0 0

**T u m o r V o lu m e ( m m 3 )**

3 0 0

2 0 0

1 0 0

0

0 1 6 8 3 3 6 5 0 4 6 7 2

**T im e [ h ]**

4 0 0

**T u m o r V o lu m e ( m m 3 )**

3 0 0

2 0 0

1 0 0

0

0 1 6 8 3 3 6 5 0 4 6 7 2

**T im e [ h ]**

4 0 0

**T u m o r V o lu m e ( m m 3 )**

3 0 0

2 0 0

1 0 0

0

0 1 6 8 3 3 6 5 0 4 6 7 2

**T im e [ h ]**

1 0 0 0

**T u m o r V o lu m e ( m m 3 )**

5 0 0

0

0 1 6 8 3 3 6 5 0 4 6 7 2

**T im e [ h ]**

4 0 0

**T u m o r V o lu m e ( m m 3 )**

3 0 0

2 0 0

1 0 0

0

0 1 6 8 3 3 6 5 0 4 6 7 2

**T im e [ h ]**

4 0 0

**T u m o r V o lu m e ( m m 3 )**

3 0 0

2 0 0

1 0 0

0

0 1 6 8 3 3 6 5 0 4 6 7 2

**T im e [ h ]**

4 0 0

**T u m o r V o lu m e ( m m 3 )**

3 0 0

2 0 0

1 0 0

0

0 1 6 8 3 3 6 5 0 4 6 7 2

**T im e [ h ]**

**C U C R C 0 2 6 C o m b in a t io n**

4 0 0

**T u m o r V o lu m e ( m m 3 )**

3 0 0

2 0 0

1 0 0

0

0 1 6 8 3 3 6 5 0 4 6 7 2

**T im e [ h ]**

4 0 0

**T u m o r V o lu m e ( m m 3 )**

3 0 0

2 0 0

1 0 0

0

0 1 6 8 3 3 6 5 0 4 6 7 2

**T im e [ h ]**

2 0 0 0

**T u m o r V o lu m e ( m m 3 )**

1 5 0 0

1 0 0 0

5 0 0

0

0 1 6 8 3 3 6 5 0 4 6 7 2

**T im e [ h ]**

4 0 0

**T u m o r V o lu m e ( m m 3 )**

3 0 0

2 0 0

1 0 0

0

0 1 6 8 3 3 6 5 0 4 6 7 2

**T im e [ h ]**

4 0 0

**T u m o r V o lu m e ( m m 3 )**

3 0 0

2 0 0

1 0 0

0

0 1 6 8 3 3 6 5 0 4 6 7 2

**T im e [ h ]**

1 0 0 0

**T u m o r V o lu m e ( m m 3 )**

8 0 0

6 0 0

4 0 0

2 0 0

0

0 1 6 8 3 3 6 5 0 4 6 7 2

**T im e [ h ]**

4 0 0

**T u m o r V o lu m e ( m m 3 )**

3 0 0

2 0 0

1 0 0

0

0 1 6 8 3 3 6 5 0 4 6 7 2

**T im e [ h ]**

4 0 0

**T u m o r V o lu m e ( m m 3 )**

3 0 0

2 0 0

1 0 0

0

0 1 6 8 3 3 6 5 0 4 6 7 2

**T im e [ h ]**

1 0 0 0

**T u m o r V o lu m e ( m m 3 )**

8 0 0

6 0 0

4 0 0

2 0 0

0

0 1 6 8 3 3 6 5 0 4 6 7 2

**T im e [ h ]**

4 0 0

**T u m o r V o lu m e ( m m 3 )**

3 0 0

2 0 0

1 0 0

0

0 1 6 8 3 3 6 5 0 4 6 7 2

**T im e [ h ]**

4 0 0

**T u m o r V o lu m e ( m m 3 )**

3 0 0

2 0 0

1 0 0

0

0 1 6 8 3 3 6 5 0 4 6 7 2

**T im e [ h ]**

4 0 0

**T u m o r V o lu m e ( m m 3 )**

3 0 0

2 0 0

1 0 0

0

0 1 6 8 3 3 6 5 0 4 6 7 2

**T im e [ h ]**

**C U C R C 0 3 4 C o n t r o l**

**2 0 0 0**

**1 0 0 0**

**2 0 0 0**

**1 5 0 0**

**T u m o r V o lu m e ( m m 3 )**

**8 0 0**

**1 5 0 0**

**T u m o r V o lu m e ( m m 3 )**

**1 0 0 0**

**6 0 0**

**4 0 0**

**1 0 0 0**

**5 0 0**

**2 0 0**

**5 0 0**

**0**

**T u m o r V o lu m e ( m m 3 )**

**0 1 6 8 3 3 6 5 0 4 6 7 2 8 4 0**

**T im e [ h ]**

**0**

**0 1 6 8 3 3 6 5 0 4 6 7 2 8 4 0**

**T im e [ h ]**

**0**

0 1 6 8 3 3 6 5 0 4 6 7 2 8 4 0

**T im e [ h ]**

**2 0 0 0**

**1 0 0 0**

**T u m o r V o lu m e ( m m 3 )**

**4 0 0**

**1 5 0 0**

**T u m o r V o lu m e ( m m 3 )**

**8 0 0**

**3 0 0**

**T u m o r V o lu m e ( m m 3 )**

**1 0 0 0**

**6 0 0**

**4 0 0**

**2 0 0**

**5 0 0**

**2 0 0**

**1 0 0**

**0**

**0 1 6 8 3 3 6 5 0 4 6 7 2 8 4 0**

**T im e [ h ]**

**0**

0 1 6 8 3 3 6 5 0 4 6 7 2 8 4 0

**T im e [ h ]**

**0**

0 1 6 8 3 3 6 5 0 4 6 7 2 8 4 0

**T im e [ h ]**

**C U C R C 0 3 4 C e tu x im a b**

**0**

**1 6 8**

**3 3 6**

**5 0 4**

**6 7 2**

**8 4 0**

4 0 0

**4 0 0**

**4 0 0**

**3 0 0**

**T u m o r V o lu m e ( m m 3 )**

**3 0 0**

**3 0 0**

**2 0 0**

**2 0 0**

**2 0 0**

**1 0 0**

**1 0 0**

**1 0 0**

**0**

**0 1 6 8 3 3 6 5 0 4 6 7 2 8 4 0**

**T im e [ h ]**

**0**

**T u m o r V o lu m e ( m m 3 )**

**0 1 6 8 3 3 6 5 0 4 6 7 2 8 4 0**

**T im e [ h ]**

**0**

**T u m o r V o lu m e ( m m 3 )**

**T im e [ h ]**

**0**

**1 6 8**

**3 3 6**

**5 0 4**

**6 7 2**

**8 4 0**

**4 0 0**

**4 0 0**

**4 0 0**

**3 0 0**

**T u m o r V o lu m e ( m m 3 )**

**3 0 0**

**3 0 0**

**2 0 0**

**2 0 0**

**2 0 0**

**1 0 0**

**1 0 0**

**1 0 0**

**0**

**T u m o r V o lu m e ( m m 3 )**

**0 1 6 8 3 3 6 5 0 4 6 7 2 8 4 0**

**T im e [ h ]**

**0**

0 1 6 8 3 3 6 5 0 4 6 7 2 8 4 0

**T im e [ h ]**

**0**

**T u m o r V o lu m e ( m m 3 )**

**T im e [ h ]**

**4 0 0**

**4 0 0**

**3 0 0**

**T u m o r V o lu m e ( m m 3 )**

**3 0 0**

**2 0 0**

**2 0 0**

**1 0 0**

**1 0 0**

**0**

**T u m o r V o lu m e ( m m 3 )**

**0 1 6 8 3 3 6 5 0 4 6 7 2 8 4 0**

**T im e [ h ]**

**0**

0 1 6 8 3 3 6 5 0 4 6 7 2 8 4 0

**T im e [ h ]**

**C U C R C 0 3 4 A l is e r t ib**

**4 0 0**

**4 0 0**

**4 0 0**

**3 0 0**

**T u m o r V o lu m e ( m m 3 )**

**3 0 0**

**3 0 0**

**2 0 0**

**2 0 0**

**2 0 0**

**1 0 0**

**1 0 0**

**1 0 0**

**0**

**T u m o r V o lu m e ( m m 3 )**

**T u m o r V o lu m e ( m m 3 )**

**0 1 6 8 3 3 6 5 0 4 6 7 2 8 4 0**

**T im e [ h ]**

**0**

0 1 6 8 3 3 6 5 0 4 6 7 2 8 4 0

**T im e [ h ]**

**0**

0 1 6 8 3 3 6 5 0 4 6 7 2 8 4 0

**T im e [ h ]**

**4 0 0**

**1 0 0 0**

**4 0 0**

**3 0 0**

**T u m o r V o lu m e ( m m 3 )**

**8 0 0**

**3 0 0**

**T u m o r V o lu m e ( m m 3 )**

**2 0 0**

**6 0 0**

**4 0 0**

**2 0 0**

**1 0 0**

**2 0 0**

**1 0 0**

**0**

**T u m o r V o lu m e ( m m 3 )**

**0 1 6 8 3 3 6 5 0 4 6 7 2 8 4 0**

**T im e [ h ]**

**0**

0 1 6 8 3 3 6 5 0 4 6 7 2 8 4 0

**T im e [ h ]**

**0**

0 1 6 8 3 3 6 5 0 4 6 7 2 8 4 0

**T im e [ h ]**

**C U C R C 0 3 4 C o m b in a t io n**

4 0 0

**4 0 0**

**4 0 0**

**3 0 0**

**T u m o r V o lu m e ( m m 3 )**

**3 0 0**

**3 0 0**

**2 0 0**

**2 0 0**

**2 0 0**

**1 0 0**

**1 0 0**

**1 0 0**

**0**

**T u m o r V o lu m e ( m m 3 )**

**T u m o r V o lu m e ( m m 3 )**

**0 1 6 8 3 3 6 5 0 4 6 7 2 8 4 0**

**T im e [ h ]**

**0**

0 1 6 8 3 3 6 5 0 4 6 7 2 8 4 0

**T im e [ h ]**

**0**

0 1 6 8 3 3 6 5 0 4 6 7 2 8 4 0

**T im e [ h ]**

**0**

**1 6 8**

**3 3 6**

**5 0 4**

**6 7 2**

**8 4 0**

**1 5 0 0**

**4 0 0**

**4 0 0**

**1 0 0 0**

**T u m o r V o lu m e ( m m 3 )**

**3 0 0**

**3 0 0**

**2 0 0**

**2 0 0**

**5 0 0**

**1 0 0**

**1 0 0**

**0**

**0 1 6 8 3 3 6 5 0 4 6 7 2 8 4 0**

**T im e [ h ]**

**0**

**T u m o r V o lu m e ( m m 3 )**

**0 1 6 8 3 3 6 5 0 4 6 7 2 8 4 0**

**T im e [ h ]**

**0**

**T u m o r V o lu m e ( m m 3 )**

**T im e [ h ]**

**4 0 0**

**4 0 0**

**4 0 0**

**3 0 0**

**T u m o r V o lu m e ( m m 3 )**

**3 0 0**

**3 0 0**

**2 0 0**

**2 0 0**

**2 0 0**

**1 0 0**

**1 0 0**

**1 0 0**

**0**

**T u m o r V o lu m e ( m m 3 )**

**T u m o r V o lu m e ( m m 3 )**

**0 1 6 8 3 3 6 5 0 4 6 7 2 8 4 0**

**T im e [ h ]**

**0**

0 1 6 8 3 3 6 5 0 4 6 7 2 8 4 0

**T im e [ h ]**

**0**

0 1 6 8 3 3 6 5 0 4 6 7 2 8 4 0

**T im e [ h ]**

**C U C R C 0 4 7 C o n t r o l**

1 5 0 0

**T u m o r V o lu m e ( m m 3 )**

1 0 0 0

5 0 0

4 0 0

**T u m o r V o lu m e ( m m 3 )**

3 0 0

2 0 0

1 0 0

4 0 0

**T u m o r V o lu m e ( m m 3 )**

3 0 0

2 0 0

1 0 0

4 0 0

**T u m o r V o lu m e ( m m 3 )**

3 0 0

2 0 0

1 0 0

0

0 1 6 8 3 3 6 5 0 4 6 7 2 8 4 0 1 0 0 8

**T im e [ h ]**

0

0 1 6 8 3 3 6 5 0 4 6 7 2 8 4 0 1 0 0 8

**T im e [ h ]**

0

0 1 6 8 3 3 6 5 0 4 6 7 2 8 4 0 1 0 0 8

**T im e [ h ]**

0

0 1 6 8 3 3 6 5 0 4 6 7 2 8 4 0 1 0 0 8

**T im e [ h ]**

1 5 0 0

**T u m o r V o lu m e ( m m 3 )**

1 0 0 0

5 0 0

0

0 1 6 8 3 3 6 5 0 4 6 7 2 8 4 0 1 0 0 8

**T im e [ h ]**

2 0 0 0

**T u m o r V o lu m e ( m m 3 )**

1 0 0 0

0

0 1 6 8 3 3 6 5 0 4 6 7 2 8 4 0 1 0 0 8

**T im e [ h ]**

1 5 0 0

**T u m o r V o lu m e ( m m 3 )**

1 0 0 0

5 0 0

0

0 1 6 8 3 3 6 5 0 4 6 7 2 8 4 0 1 0 0 8

**T im e [ h ]**

4 0 0

**T u m o r V o lu m e ( m m 3 )**

3 0 0

2 0 0

1 0 0

0

0 1 6 8 3 3 6 5 0 4 6 7 2 8 4 0 1 0 0 8

**T im e [ h ]**

4 0 0

**T u m o r V o lu m e ( m m 3 )**

3 0 0

2 0 0

1 0 0

0

0 1 6 8 3 3 6 5 0 4 6 7 2 8 4 0 1 0 0 8

**T im e [ h ]**

1 5 0 0

**T u m o r V o lu m e ( m m 3 )**

1 0 0 0

5 0 0

0

0 1 6 8 3 3 6 5 0 4 6 7 2 8 4 0 1 0 0 8

**T im e [ h ]**

2 0 0 0

**T u m o r V o lu m e ( m m 3 )**

1 0 0 0

0

0 1 6 8 3 3 6 5 0 4 6 7 2 8 4 0 1 0 0 8

**T im e [ h ]**

1 5 0 0

**T u m o r V o lu m e ( m m 3 )**

1 0 0 0

5 0 0

0

0 1 6 8 3 3 6 5 0 4 6 7 2 8 4 0 1 0 0 8

**T im e [ h ]**

1 0 0 0

**T u m o r V o lu m e ( m m 3 )**

8 0 0

6 0 0

4 0 0

2 0 0

0

0 1 6 8 3 3 6 5 0 4 6 7 2 8 4 0 1 0 0 8

**T im e [ h ]**

1 0 0 0

**T u m o r V o lu m e ( m m 3 )**

8 0 0

6 0 0

4 0 0

2 0 0

0

0 1 6 8 3 3 6 5 0 4 6 7 2 8 4 0 1 0 0 8

**T im e [ h ]**

**C U C R C 0 4 7 C e tu x im a b**

8 0 0

**T u m o r V o lu m e ( m m 3 )**

6 0 0

4 0 0

2 0 0

0

0 1 6 8 3 3 6 5 0 4 6 7 2 8 4 0 1 0 0 8

**T im e [ h ]**

8 0 0

**T u m o r V o lu m e ( m m 3 )**

6 0 0

4 0 0

2 0 0

0

0 1 6 8 3 3 6 5 0 4 6 7 2 8 4 0 1 0 0 8

**T im e [ h ]**

8 0 0

**T u m o r V o lu m e ( m m 3 )**

6 0 0

4 0 0

2 0 0

0

0 1 6 8 3 3 6 5 0 4 6 7 2 8 4 0 1 0 0 8

**T im e [ h ]**

2 0 0 0

**T u m o r V o lu m e ( m m 3 )**

1 5 0 0

1 0 0 0

5 0 0

0

0 1 6 8 3 3 6 5 0 4 6 7 2 8 4 0 1 0 0 8

**T im e [ h ]**

8 0 0

**T u m o r V o lu m e ( m m 3 )**

6 0 0

4 0 0

2 0 0

0

0 1 6 8 3 3 6 5 0 4 6 7 2 8 4 0 1 0 0 8

**T im e [ h ]**

2 0 0 0

**T u m o r V o lu m e ( m m 3 )**

1 5 0 0

1 0 0 0

5 0 0

0

0 1 6 8 3 3 6 5 0 4 6 7 2 8 4 0 1 0 0 8

**T im e [ h ]**

2 0 0 0

**T u m o r V o lu m e ( m m 3 )**

1 5 0 0

1 0 0 0

5 0 0

0

0 1 6 8 3 3 6 5 0 4 6 7 2 8 4 0 1 0 0 8

**T im e [ h ]**

2 0 0 0

**T u m o r V o lu m e ( m m 3 )**

1 5 0 0

1 0 0 0

5 0 0

0

0 1 6 8 3 3 6 5 0 4 6 7 2 8 4 0 1 0 0 8

**T im e [ h ]**

8 0 0

**T u m o r V o lu m e ( m m 3 )**

6 0 0

4 0 0

2 0 0

0

0 1 6 8 3 3 6 5 0 4 6 7 2 8 4 0 1 0 0 8

**T im e [ h ]**

8 0 0

**T u m o r V o lu m e ( m m 3 )**

6 0 0

4 0 0

2 0 0

0

0 1 6 8 3 3 6 5 0 4 6 7 2 8 4 0 1 0 0 8

**T im e [ h ]**

2 0 0 0

**T u m o r V o lu m e ( m m 3 )**

1 5 0 0

1 0 0 0

5 0 0

0

0 1 6 8 3 3 6 5 0 4 6 7 2 8 4 0 1 0 0 8

**T im e [ h ]**

2 0 0 0

**T u m o r V o lu m e ( m m 3 )**

1 5 0 0

1 0 0 0

5 0 0

0

0 1 6 8 3 3 6 5 0 4 6 7 2 8 4 0 1 0 0 8

**T im e [ h ]**

**C U C R C 0 4 7 A l is e r t ib**

4 0 0

**T u m o r V o lu m e ( m m 3 )**

3 0 0

2 0 0

1 0 0

0

0 5 0 0 1 0 0 0

**T im e [ h ]**

1 0 0 0

**T u m o r V o lu m e ( m m 3 )**

8 0 0

6 0 0

4 0 0

2 0 0

0

0 1 6 8 3 3 6 5 0 4 6 7 2 8 4 0 1 0 0 8

**T im e [ h ]**

1 0 0 0

**T u m o r V o lu m e ( m m 3 )**

8 0 0

6 0 0

4 0 0

2 0 0

0

0 1 6 8 3 3 6 5 0 4 6 7 2 8 4 0 1 0 0 8

**T im e [ h ]**

1 0 0 0

**T u m o r V o lu m e ( m m 3 )**

8 0 0

6 0 0

4 0 0

2 0 0

0

0 1 6 8 3 3 6 5 0 4 6 7 2 8 4 0 1 0 0 8

**T im e [ h ]**

2 0 0 0

**T u m o r V o lu m e ( m m 3 )**

1 5 0 0

1 0 0 0

5 0 0

0

0 1 6 8 3 3 6 5 0 4 6 7 2 8 4 0 1 0 0 8

**T im e [ h ]**

1 0 0 0

**T u m o r V o lu m e ( m m 3 )**

8 0 0

6 0 0

4 0 0

2 0 0

0

0 1 6 8 3 3 6 5 0 4 6 7 2 8 4 0 1 0 0 8

**T im e [ h ]**

2 0 0 0

**T u m o r V o lu m e ( m m 3 )**

1 5 0 0

1 0 0 0

5 0 0

0

0 1 6 8 3 3 6 5 0 4 6 7 2 8 4 0 1 0 0 8

**T im e [ h ]**

1 0 0 0

**T u m o r V o lu m e ( m m 3 )**

8 0 0

6 0 0

4 0 0

2 0 0

0

0 1 6 8 3 3 6 5 0 4 6 7 2 8 4 0 1 0 0 8

**T im e [ h ]**

1 0 0 0

**T u m o r V o lu m e ( m m 3 )**

8 0 0

6 0 0

4 0 0

2 0 0

0

0 1 6 8 3 3 6 5 0 4 6 7 2 8 4 0 1 0 0 8

**T im e [ h ]**

2 0 0 0

**T u m o r V o lu m e ( m m 3 )**

1 5 0 0

1 0 0 0

5 0 0

0

0 1 6 8 3 3 6 5 0 4 6 7 2 8 4 0 1 0 0 8

**T im e [ h ]**

3 0 0 0

**T u m o r V o lu m e ( m m 3 )**

2 0 0 0

1 0 0 0

0

0 1 6 8 3 3 6 5 0 4 6 7 2 8 4 0 1 0 0 8

**T im e [ h ]**

3 0 0 0

**T u m o r V o lu m e ( m m 3 )**

2 0 0 0

1 0 0 0

0

0 1 6 8 3 3 6 5 0 4 6 7 2 8 4 0 1 0 0 8

**T im e [ h ]**

2 0 0 0

**T u m o r V o lu m e ( m m 3 )**

1 5 0 0

1 0 0 0

5 0 0

0

0 1 6 8 3 3 6 5 0 4 6 7 2 8 4 0 1 0 0 8

**T im e [ h ]**

**C U C R C 0 4 7 C o m b in a t io n**

1 0 0 0

**T u m o r V o lu m e ( m m 3 )**

8 0 0

6 0 0

4 0 0

2 0 0

0

0 1 6 8 3 3 6 5 0 4 6 7 2 8 4 0 1 0 0 8

**T im e [ h ]**

1 0 0 0

**T u m o r V o lu m e ( m m 3 )**

8 0 0

6 0 0

4 0 0

2 0 0

0

0 1 6 8 3 3 6 5 0 4 6 7 2 8 4 0 1 0 0 8

**T im e [ h ]**

2 0 0 0

**T u m o r V o lu m e ( m m 3 )**

1 5 0 0

1 0 0 0

5 0 0

0

0 1 6 8 3 3 6 5 0 4 6 7 2 8 4 0 1 0 0 8

**T im e [ h ]**

2 0 0 0

**T u m o r V o lu m e ( m m 3 )**

1 5 0 0

1 0 0 0

5 0 0

0

0 1 6 8 3 3 6 5 0 4 6 7 2 8 4 0 1 0 0 8

**T im e [ h ]**

1 5 0 0

**T u m o r V o lu m e ( m m 3 )**

1 0 0 0

5 0 0

0

0 1 6 8 3 3 6 5 0 4 6 7 2 8 4 0 1 0 0 8

**T im e [ h ]**

1 5 0 0

**T u m o r V o lu m e ( m m 3 )**

1 0 0 0

5 0 0

0

0 1 6 8 3 3 6 5 0 4 6 7 2 8 4 0 1 0 0 8

**T im e [ h ]**

1 5 0 0

**T u m o r V o lu m e ( m m 3 )**

1 0 0 0

5 0 0

0

0 1 6 8 3 3 6 5 0 4 6 7 2 8 4 0 1 0 0 8

**T im e [ h ]**

1 0 0 0

**T u m o r V o lu m e ( m m 3 )**

8 0 0

6 0 0

4 0 0

2 0 0

0

0 1 6 8 3 3 6 5 0 4 6 7 2 8 4 0 1 0 0 8

**T im e [ h ]**

4 0 0

**T u m o r V o lu m e ( m m 3 )**

3 0 0

2 0 0

1 0 0

0

0 1 6 8 3 3 6 5 0 4 6 7 2 8 4 0 1 0 0 8

**T im e [ h ]**

1 0 0 0

**T u m o r V o lu m e ( m m 3 )**

8 0 0

6 0 0

4 0 0

2 0 0

0

0 1 6 8 3 3 6 5 0 4 6 7 2 8 4 0 1 0 0 8

**T im e [ h ]**

1 0 0 0

**T u m o r V o lu m e ( m m 3 )**

8 0 0

6 0 0

4 0 0

2 0 0

0

0 1 6 8 3 3 6 5 0 4 6 7 2 8 4 0 1 0 0 8

**T im e [ h ]**

2 0 0 0

**T u m o r V o lu m e ( m m 3 )**

1 5 0 0

1 0 0 0

5 0 0

0

0 1 6 8 3 3 6 5 0 4 6 7 2 8 4 0 1 0 0 8

**T im e [ h ]**

**C U C R C 1 2 5 C o n t r o l**

1 5 0 0

**T u m o r V o lu m e ( m m 3 )**

1 0 0 0

5 0 0

0

0 1 6 8 3 3 6 5 0 4 6 7 2 8 4 0 1 0 0 8

**T im e [ h ]**

1 5 0 0

**T u m o r V o lu m e ( m m 3 )**

1 0 0 0

5 0 0

0

0 1 6 8 3 3 6 5 0 4 6 7 2 8 4 0 1 0 0 8

**T im e [ h ]**

1 5 0 0

**T u m o r V o lu m e ( m m 3 )**

1 0 0 0

5 0 0

0

0 1 6 8 3 3 6 5 0 4 6 7 2 8 4 0 1 0 0 8

**T im e [ h ]**

1 5 0 0

**T u m o r V o lu m e ( m m 3 )**

1 0 0 0

5 0 0

0

0 1 6 8 3 3 6 5 0 4 6 7 2 8 4 0 1 0 0 8

**T im e [ h ]**

2 5 0 0

**T u m o r V o lu m e ( m m 3 )**

2 0 0 0

1 5 0 0

1 0 0 0

5 0 0

0

0 1 6 8 3 3 6 5 0 4 6 7 2 8 4 0 1 0 0 8

**T im e [ h ]**

2 5 0 0

**T u m o r V o lu m e ( m m 3 )**

2 0 0 0

1 5 0 0

1 0 0 0

5 0 0

0

0 1 6 8 3 3 6 5 0 4 6 7 2 8 4 0 1 0 0 8

**T im e [ h ]**

2 5 0 0

**T u m o r V o lu m e ( m m 3 )**

2 0 0 0

1 5 0 0

1 0 0 0

5 0 0

0

0 1 6 8 3 3 6 5 0 4 6 7 2 8 4 0 1 0 0 8

**T im e [ h ]**

1 5 0 0

**T u m o r V o lu m e ( m m 3 )**

1 0 0 0

5 0 0

0

0 1 6 8 3 3 6 5 0 4 6 7 2 8 4 0 1 0 0 8

**T im e [ h ]**

1 5 0 0

**T u m o r V o lu m e ( m m 3 )**

1 0 0 0

5 0 0

0

0 1 6 8 3 3 6 5 0 4 6 7 2 8 4 0 1 0 0 8

**T im e [ h ]**

2 5 0 0

**T u m o r V o lu m e ( m m 3 )**

2 0 0 0

1 5 0 0

1 0 0 0

5 0 0

0

0 1 6 8 3 3 6 5 0 4 6 7 2 8 4 0 1 0 0 8

**T im e [ h ]**

**C U C R C 1 2 5 C e tu x im a b**

4 0 0

**T u m o r V o lu m e ( m m 3 )**

3 0 0

2 0 0

1 0 0

0

0 1 6 8 3 3 6 5 0 4 6 7 2 8 4 0 1 0 0 8

**T im e [ h ]**

8 0 0

**T u m o r V o lu m e ( m m 3 )**

6 0 0

4 0 0

2 0 0

0

0 1 6 8 3 3 6 5 0 4 6 7 2 8 4 0 1 0 0 8

**T im e [ h ]**

4 0 0

**T u m o r V o lu m e ( m m 3 )**

3 0 0

2 0 0

1 0 0

0

0 1 6 8 3 3 6 5 0 4 6 7 2 8 4 0 1 0 0 8

**T im e [ h ]**

4 0 0

**T u m o r V o lu m e ( m m 3 )**

3 0 0

2 0 0

1 0 0

0

0 1 6 8 3 3 6 5 0 4 6 7 2 8 4 0 1 0 0 8

**T im e [ h ]**

8 0 0

**T u m o r V o lu m e ( m m 3 )**

6 0 0

4 0 0

2 0 0

0

0 1 6 8 3 3 6 5 0 4 6 7 2 8 4 0 1 0 0 8

**T im e [ h ]**

4 0 0

**T u m o r V o lu m e ( m m 3 )**

3 0 0

2 0 0

1 0 0

0

0 1 6 8 3 3 6 5 0 4 6 7 2 8 4 0 1 0 0 8

**T im e [ h ]**

4 0 0

**T u m o r V o lu m e ( m m 3 )**

3 0 0

2 0 0

1 0 0

0

0 1 6 8 3 3 6 5 0 4 6 7 2 8 4 0 1 0 0 8

**T im e [ h ]**

8 0 0

**T u m o r V o lu m e ( m m 3 )**

6 0 0

4 0 0

2 0 0

0

0 1 6 8 3 3 6 5 0 4 6 7 2 8 4 0 1 0 0 8

**T im e [ h ]**

1 5 0 0

T u m o r V o lu m e ( m m 3 )

1 0 0 0

5 0 0

0

0 1 6 8 3 3 6 5 0 4 6 7 2 8 4 0 1 0 0 8

T im e [ h ]

4 0 0

**T u m o r V o lu m e ( m m 3 )**

3 0 0

2 0 0

1 0 0

0

0 1 6 8 3 3 6 5 0 4 6 7 2 8 4 0 1 0 0 8

**T im e [ h ]**

4 0 0

**T u m o r V o lu m e ( m m 3 )**

3 0 0

2 0 0

1 0 0

0

0 1 6 8 3 3 6 5 0 4 6 7 2 8 4 0 1 0 0 8

**T im e [ h ]**

4 0 0

**T u m o r V o lu m e ( m m 3 )**

3 0 0

2 0 0

1 0 0

0

0 1 6 8 3 3 6 5 0 4 6 7 2 8 4 0 1 0 0 8

**T im e [ h ]**

8 0 0

**T u m o r V o lu m e ( m m 3 )**

6 0 0

4 0 0

2 0 0

0

0 1 6 8 3 3 6 5 0 4 6 7 2 8 4 0 1 0 0 8

**T im e [ h ]**

**C U C R C 1 2 5 A l is e r t ib**

4 0 0

**T u m o r V o lu m e ( m m 3 )**

3 0 0

2 0 0

1 0 0

0

0 1 6 8 3 3 6 5 0 4 6 7 2 8 4 0 1 0 0 8

**T im e [ h ]**

1 0 0 0

**T u m o r V o lu m e ( m m 3 )**

8 0 0

6 0 0

4 0 0

2 0 0

0

0 1 6 8 3 3 6 5 0 4 6 7 2 8 4 0 1 0 0 8

**T im e [ h ]**

2 0 0 0

**T u m o r V o lu m e ( m m 3 )**

1 5 0 0

1 0 0 0

5 0 0

0

0 1 6 8 3 3 6 5 0 4 6 7 2 8 4 0 1 0 0 8

**T im e [ h ]**

4 0 0

**T u m o r V o lu m e ( m m 3 )**

3 0 0

2 0 0

1 0 0

0

0 1 6 8 3 3 6 5 0 4 6 7 2 8 4 0 1 0 0 8

**T im e [ h ]**

1 0 0 0

**T u m o r V o lu m e ( m m 3 )**

8 0 0

6 0 0

4 0 0

2 0 0

0

0 1 6 8 3 3 6 5 0 4 6 7 2 8 4 0 1 0 0 8

**T im e [ h ]**

1 0 0 0

**T u m o r V o lu m e ( m m 3 )**

8 0 0

6 0 0

4 0 0

2 0 0

0

0 1 6 8 3 3 6 5 0 4 6 7 2 8 4 0 1 0 0 8

**T im e [ h ]**

1 0 0 0

**T u m o r V o lu m e ( m m 3 )**

8 0 0

6 0 0

4 0 0

2 0 0

0

0 1 6 8 3 3 6 5 0 4 6 7 2 8 4 0 1 0 0 8

**T im e [ h ]**

1 0 0 0

**T u m o r V o lu m e ( m m 3 )**

8 0 0

6 0 0

4 0 0

2 0 0

0

0 1 6 8 3 3 6 5 0 4 6 7 2 8 4 0 1 0 0 8

**T im e [ h ]**

1 0 0 0

**T u m o r V o lu m e ( m m 3 )**

8 0 0

6 0 0

4 0 0

2 0 0

0

0 1 6 8 3 3 6 5 0 4 6 7 2 8 4 0 1 0 0 8

**T im e [ h ]**

2 0 0 0

**T u m o r V o lu m e ( m m 3 )**

1 5 0 0

1 0 0 0

5 0 0

0

0 1 6 8 3 3 6 5 0 4 6 7 2 8 4 0 1 0 0 8

**T im e [ h ]**

4 0 0

**T u m o r V o lu m e ( m m 3 )**

3 0 0

2 0 0

1 0 0

0

0 1 6 8 3 3 6 5 0 4 6 7 2 8 4 0 1 0 0 8

**T im e [ h ]**

**C U C R C 1 2 5 C o m b in a t io n**

4 0 0

**T u m o r V o lu m e ( m m 3 )**

3 0 0

2 0 0

1 0 0

0

0 1 6 8 3 3 6 5 0 4 6 7 2 8 4 0 1 0 0 8

**T im e [ h ]**

4 0 0

**T u m o r V o lu m e ( m m 3 )**

3 0 0

2 0 0

1 0 0

0

0 1 6 8 3 3 6 5 0 4 6 7 2 8 4 0 1 0 0 8

**T im e [ h ]**

4 0 0

**T u m o r V o lu m e ( m m 3 )**

3 0 0

2 0 0

1 0 0

0

0 1 6 8 3 3 6 5 0 4 6 7 2 8 4 0 1 0 0 8

**T im e [ h ]**

4 0 0

**T u m o r V o lu m e ( m m 3 )**

3 0 0

2 0 0

1 0 0

0

0 1 6 8 3 3 6 5 0 4 6 7 2 8 4 0 1 0 0 8

**T im e [ h ]**

4 0 0

**T u m o r V o lu m e ( m m 3 )**

3 0 0

2 0 0

1 0 0

0

0 1 6 8 3 3 6 5 0 4 6 7 2 8 4 0 1 0 0 8

**T im e [ h ]**

4 0 0

**T u m o r V o lu m e ( m m 3 )**

3 0 0

2 0 0

1 0 0

0

0 1 6 8 3 3 6 5 0 4 6 7 2 8 4 0 1 0 0 8

**T im e [ h ]**

4 0 0

**T u m o r V o lu m e ( m m 3 )**

3 0 0

2 0 0

1 0 0

0

0 1 6 8 3 3 6 5 0 4 6 7 2 8 4 0 1 0 0 8

**T im e [ h ]**

6 0 0

**T u m o r V o lu m e ( m m 3 )**

4 0 0

2 0 0

0

0 1 6 8 3 3 6 5 0 4 6 7 2 8 4 0 1 0 0 8

**T im e [ h ]**

4 0 0

**T u m o r V o lu m e ( m m 3 )**

3 0 0

2 0 0

1 0 0

0

0 1 6 8 3 3 6 5 0 4 6 7 2 8 4 0 1 0 0 8

**T im e [ h ]**

4 0 0

**T u m o r V o lu m e ( m m 3 )**

3 0 0

2 0 0

1 0 0

0

0 1 6 8 3 3 6 5 0 4 6 7 2 8 4 0 1 0 0 8

**T im e [ h ]**

6 0 0

**T u m o r V o lu m e ( m m 3 )**

4 0 0

2 0 0

0

0 1 6 8 3 3 6 5 0 4 6 7 2 8 4 0 1 0 0 8

**T im e [ h ]**

6 0 0

**T u m o r V o lu m e ( m m 3 )**

4 0 0

2 0 0

0

0 1 6 8 3 3 6 5 0 4 6 7 2 8 4 0 1 0 0 8

**T im e [ h ]**

1 0 0 0

**T u m o r V o lu m e ( m m 3 )**

8 0 0

6 0 0

4 0 0

2 0 0

0

0 1 6 8 3 3 6 5 0 4 6 7 2 8 4 0 1 0 0 8

**T im e [ h ]**

6 0 0

**T u m o r V o lu m e ( m m 3 )**

4 0 0

2 0 0

0

0 1 6 8 3 3 6 5 0 4 6 7 2 8 4 0 1 0 0 8

**T im e [ h ]**

6 0 0

**T u m o r V o lu m e ( m m 3 )**

4 0 0

2 0 0

0

0 1 6 8 3 3 6 5 0 4 6 7 2 8 4 0 1 0 0 8

**T im e [ h ]**
